# Supplementary material for: Trust within human-machine collectives depends on the perceived consensus about cooperative norms
Source: Nat Commun. 2023 May 30;14:3108. doi: 10.1038/s41467-023-38592-5 (PMC10229533; doi:10.1038/s41467-023-38592-5)
Supplement: Supplementary file 1 — Supplementary Information [file 41467_2023_38592_MOESM1_ESM.pdf]

Supplementary Information

Trust within human-machine collectives depends on the perceived consensus about cooperative norms

Kinga Makovi, Anahit Sargsyan, Wendi Li, Jean-François Bonnefon, Talal Rahwan*

*E-mail: talal.rahwan@nyu.edu

Contents

Supplementary Figures **3**

Supplementary Note 1: Robustness Analysis of Study 3 **8**

Supplementary Note 2: Robustness Analysis of Study 4 **12**

Supplementary Note 3: Robustness Analysis of Study 5 **14**

Supplementary Note 4: Complete Regression Tables for Study 3 **17**

Supplementary Note 5: Sample Composition of Study 1–5 **19**

Supplementary Note 6: Comparing Samples Across Studies 2–5 **20**

Supplementary Note 7: Contrasting Study 4 and Study 5 **22**

Supplementary Note 8: Justifications for Helping and Trust Decisions **29**

Supplementary Note 9: Comparing the Distribution of Trust-gain in Study 2 and Study 3 **45**

Supplementary Note 10: Analyses of Selective Non-response 48

Supplementary Figures

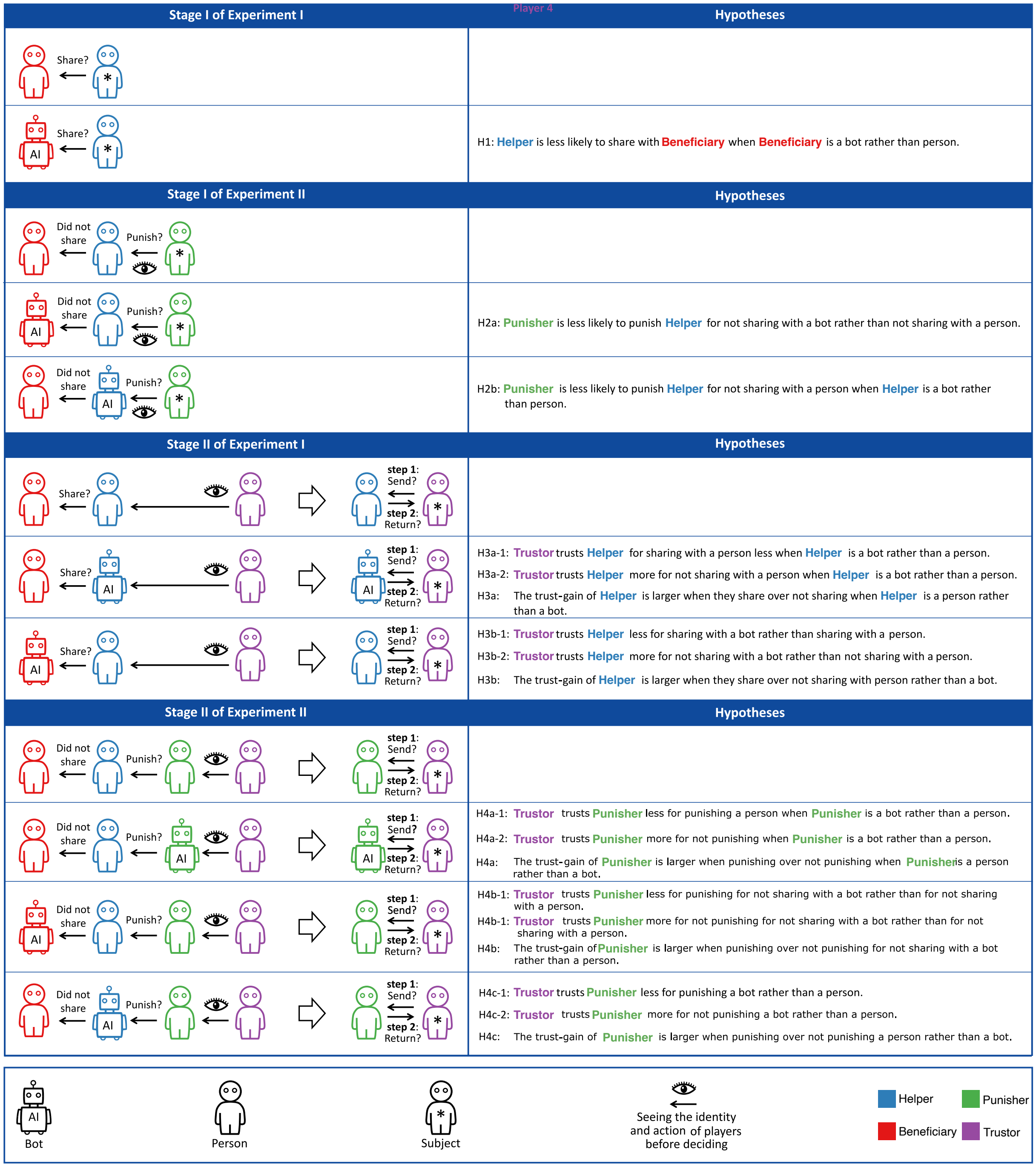

Supplementary Figure S1: **The experimental conditions of Study 2, and the hypotheses associated with each condition reflected in the pre-analysis plan and amendment.** These hypotheses map on the research questions formulated in the paper. Specifically H1 corresponds to **B1**; H2a to **B3**; H2b to **H2**; H3a-1, H3a-2 and H3a to **H1**; H3b-1, H3b-2 and H3b to **B2**; H4a-1, H4a-2 and H4a to **P1**; H4b-1, H4b-2 and H4b to **B4**; H4c-1, H4c-2 and H4c to **H3**.

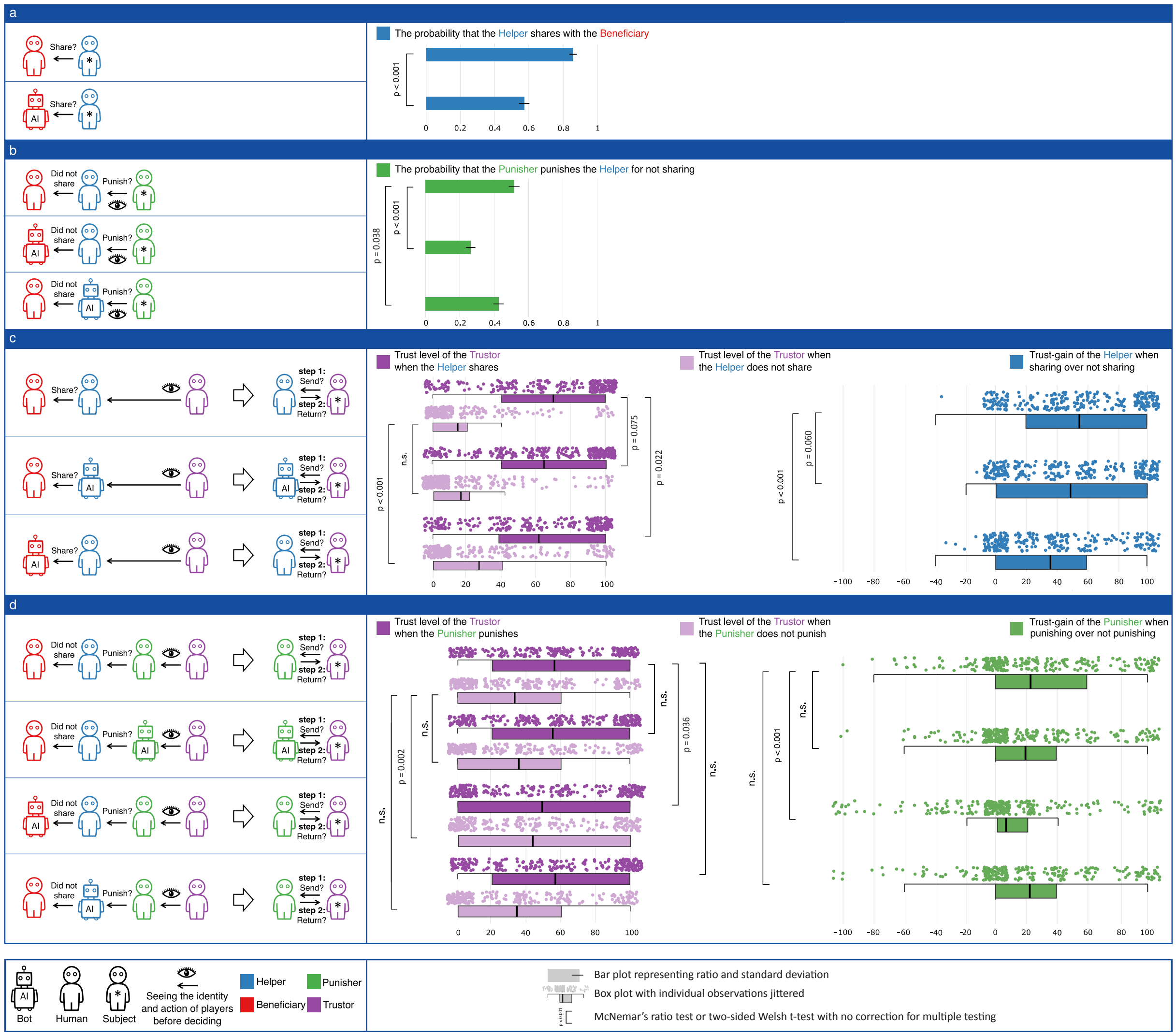

Supplementary Figure S2: **Results of Study 2 restricted to people who have answered all the comprehension check questions correctly.** In panel (a) the number of participants is, 268 and 269 corresponding to the plotted order of bars from top to bottom. In panel (b) the number of participants is 264, 276 and 275 corresponding to the plotted order of bars. In panel (c) the number of participants is 272, 265 and 285 corresponding to the plotted order of bars. In panel (d) the number of participants is 268, 267, 280 and 262 corresponding to the plotted order of bars.

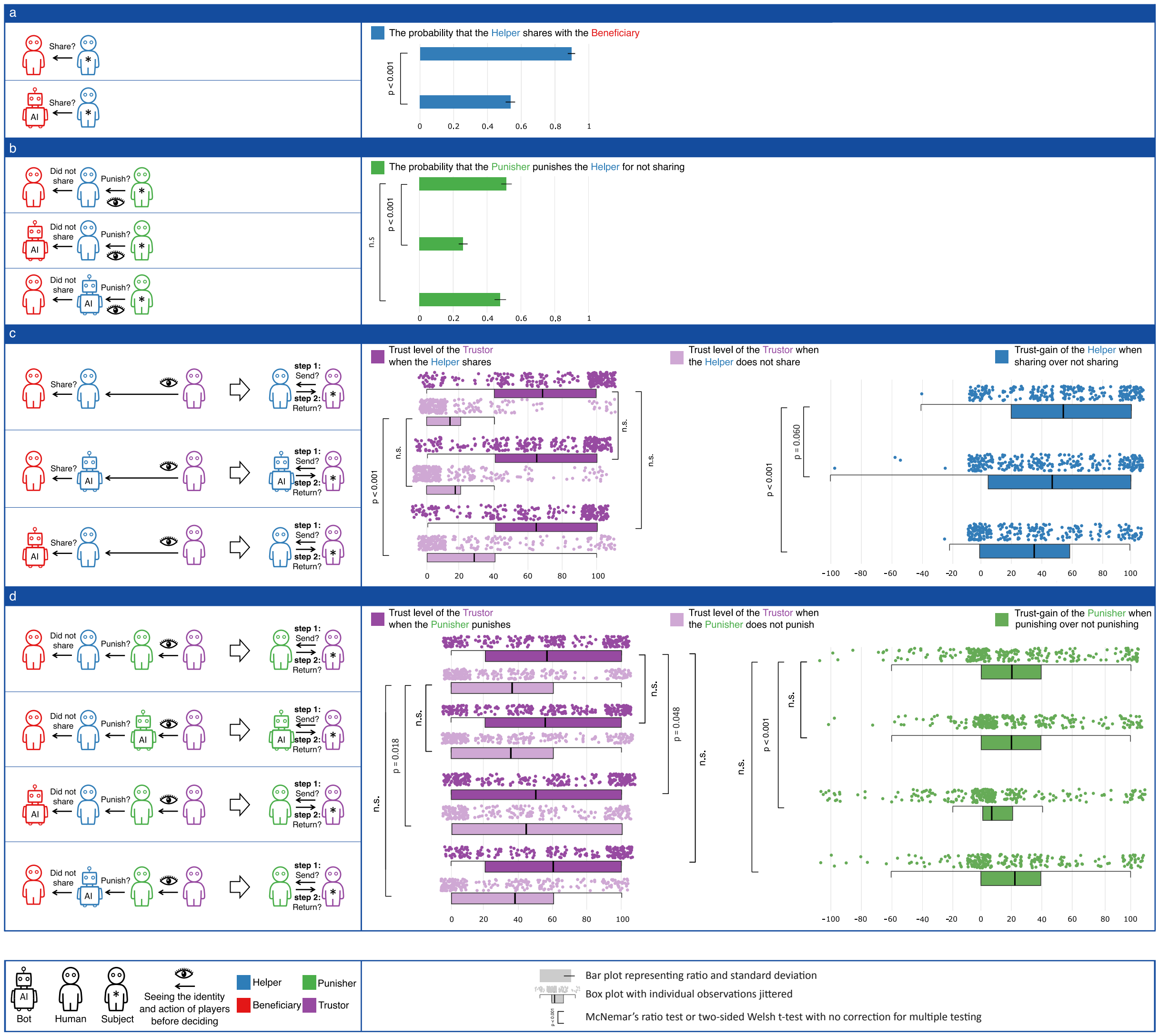

Supplementary Figure S3: **Results of Study 2 restricted to people who passed the manipulation check.** In panel (a) the number of participants is 246 and 253 corresponding to the plotted order of bars from top to bottom. In panel (b) the number of participants is 246, 253 and 243 corresponding to the plotted order of bars. In panel (c) the number of participants is 280, 262 and 298 corresponding to the plotted order of bars. In panel (d) the number of participants is 280, 291, 280 and 239 corresponding to the plotted order of bars.

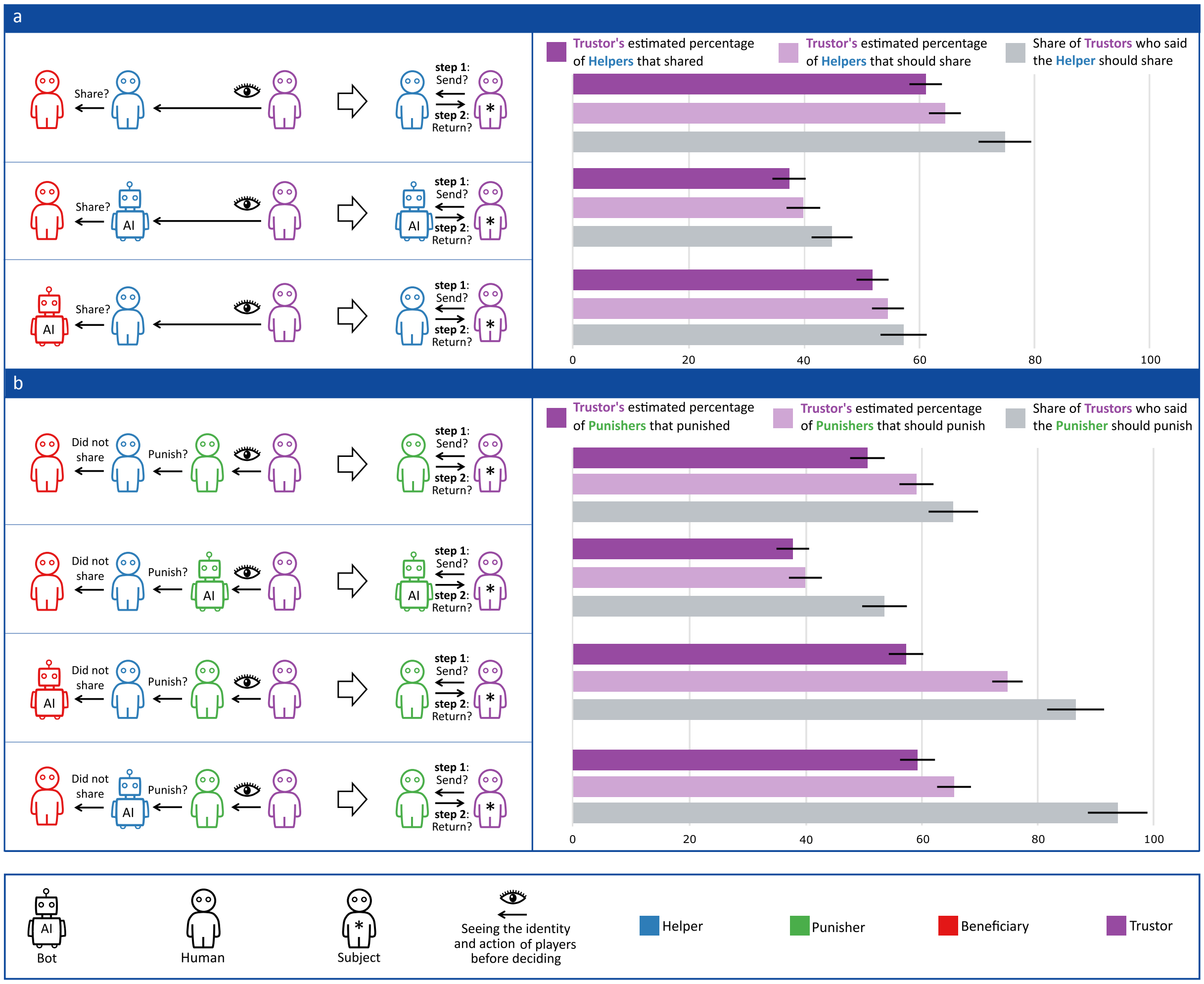

Supplementary Figure S4: **Empirical and normative expectations for sharing and third-party punishment determined in Study 3.** In panel (a) the number of participants is 355, 358 and 362 for each experimental condition. In panel (b) the number of participants is 361, 359, 359 and 360 for each experimental condition.

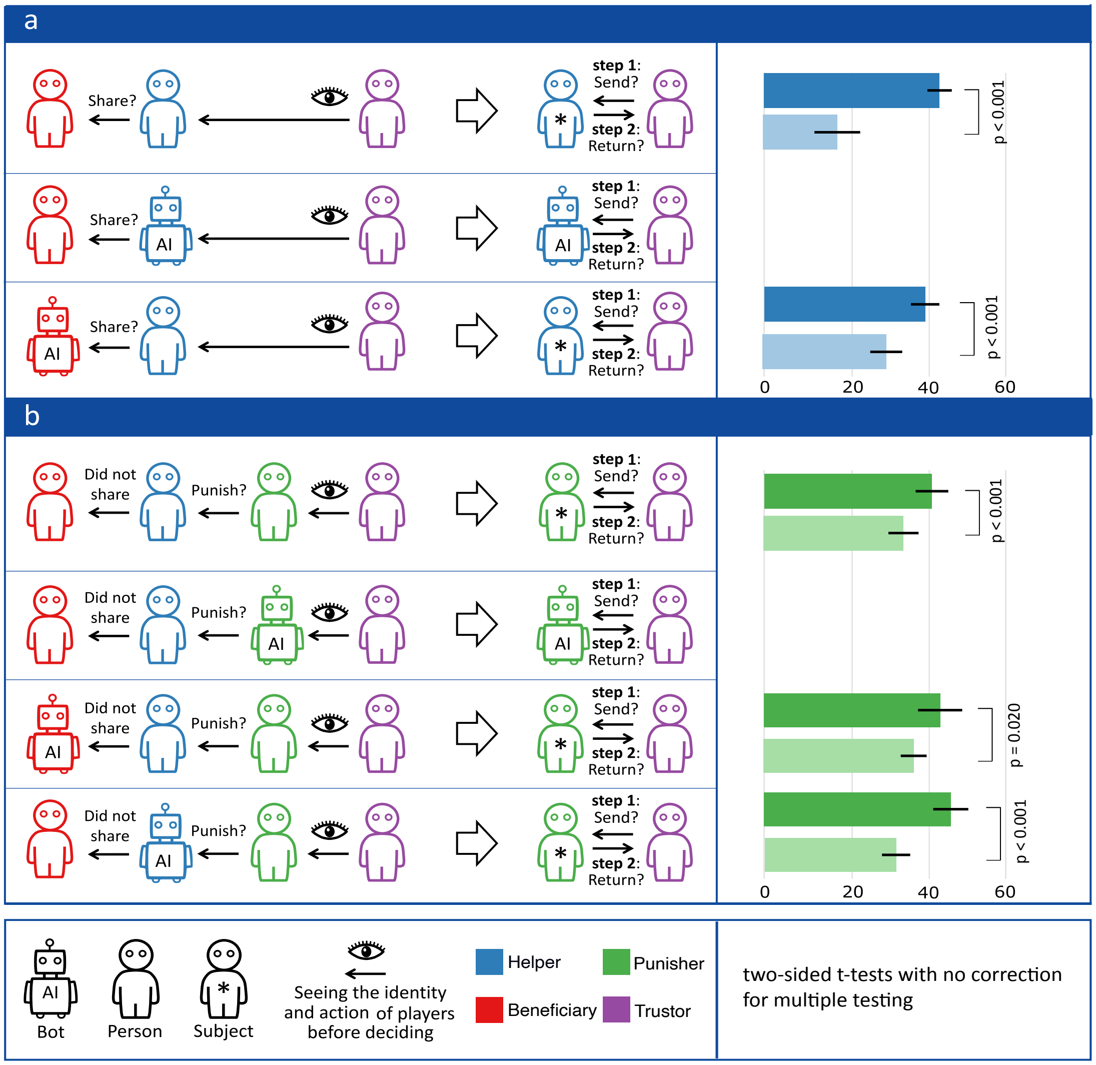

Supplementary Figure S5: **Sharing and third-party punishment is a costly signal of trustworthiness, regardless of the identity of players.** The bars quantify the average proportion returned by Helpers and Punishers in the trust game. The darker colored bars correspond to participants who shared or punished in the first stage, while lighter ones to those who didn't share or didn't punish. In panel (a) the number of participants is 271, 46, 186 and 130 corresponding to the plotted order of bars from top to bottom. In panel (b) the number of participants is 151, 165, 134, 181, 88 and 148 corresponding to the plotted order of bars.

Supplementary Note 1: Robustness Analysis of Study 3

Variables	Estimate	p-value	Estimate	p-value
Consensus	0.394	$p < 0.001$	0.187	$p = 0.096$
	(0.269, 0.518)		(-0.033, 0.408)	
Norm			✓	
Empirical expectations			✓	
Fixed effects			✓	
Controls			✓	
Observations	417		417	
Adjusted R-squared	0.083		0.095	

Supplementary Table S1: **The relationship between the Trustor's beliefs of norm-consensus over the Helper's sharing behavior and the trust that the Helper gains from sharing using multiple OLS regression among participants who answered all eight comprehension check questions correctly.** A ✓ indicates the inclusion of variables. Dependent variable: trust-gain of the Helper; Consensus: Trustor's guess of injunctive norm-consensus over the Helper's sharing; Norm: if one should share; Empirical expectations: the guessed proportion of Helpers who share; Fixed effects: fixed-effects for the experimental conditions; Controls: age, gender, race, education, income, and region. 95% confidence intervals are in parentheses.

Variables	Estimate	p-value	Estimate	p-value
Consensus	0.376	$p < 0.001$	0.252	$p = 0.001$
	(0.284, 0.467)		(0.099, 0.404)	
Norm			✓	
Empirical expectations			✓	
Fixed effects			✓	
Controls			✓	
Observations	803		803	
Adjusted R-squared	0.073		0.094	

Supplementary Table S2: **The relationship between the Trustor's beliefs of norm-consensus over the Helper's sharing behavior and the trust that the Helper gains from sharing using multiple OLS regres among participants who passed the manipulation check.** A ✓ indicates the inclusion of variables. Dependent variable: trust-gain of the Helper; Consensus: Trustor's guess of injunctive norm-consensus over the Helpers sharing; Norm: if one should share; Empirical expectations: the guessed proportion of Helpers who share; Fixed effects: fixed-effects for the experimental conditions; Controls: age, gender, race, education, income, and region. 95% confidence intervals are in parentheses.

Variables	Estimate	p-value	Estimate	p-value
Consensus	0.341	$p < 0.001$	0.119	$p = 0.001$
	(0.233, 0.448)		(-0.064, 0.301)	
Norm			✓	
Empirical expectations			✓	
Fixed effects			✓	
Controls			✓	
Observations	593		593	
Adjusted R-squared	0.060		0.094	

Supplementary Table S3: **The relationship between the Trustor's beliefs of norm-consensus over the Punisher's punishing behavior and the trust that the Punisher gains from punishing using multiple OLS regress among participants who answered all eight comprehension check questions correctly.** A ✓ indicates the inclusion of variables. Dependent variable: trust-gain of the Punisher; Consensus: Trustor's guess of injunctive norm-consensus over Punisher's punishing; Norm: if one should punish; Empirical expectations: the guessed proportion of Punishers who punish; Fixed effects: fixed-effects for the experimental conditions; Controls: age, gender, race, education, income, and region. 95% confidence intervals are in parentheses.

Variables	Estimate	p-value	Estimate	p-value
Consensus	0.265	$p < 0.001$	0.252	$p = 0.001$
	(0.182, 0.348)		(-0.064, 0.301)	
Norm			✓	
Empirical expectations			✓	
Fixed effects			✓	
Controls			✓	
Observations	1094		1094	
Adjusted R-squared	0.034		0.094	

Supplementary Table S4: **The relationship between the Trustor's beliefs of norm-consensus over the Punisher's punishing behavior and the trust that the Punisher gains from punishing using multiple OLS regress among participants who passed the manipulation check.** A ✓ indicates the inclusion of variables. Dependent variable: trust-gain of the Punisher; Consensus: Trustor's guess of injunctive norm-consensus over Punisher's punishing; Norm: if one should punish; Empirical expectations: the guessed proportion of Punishers who punish; Fixed effects: fixed-effects for the experimental conditions; Controls: age, gender, race, education, income, and region. 95% confidence intervals are in parentheses.

Supplementary Note 2: Robustness Analysis of Study 4

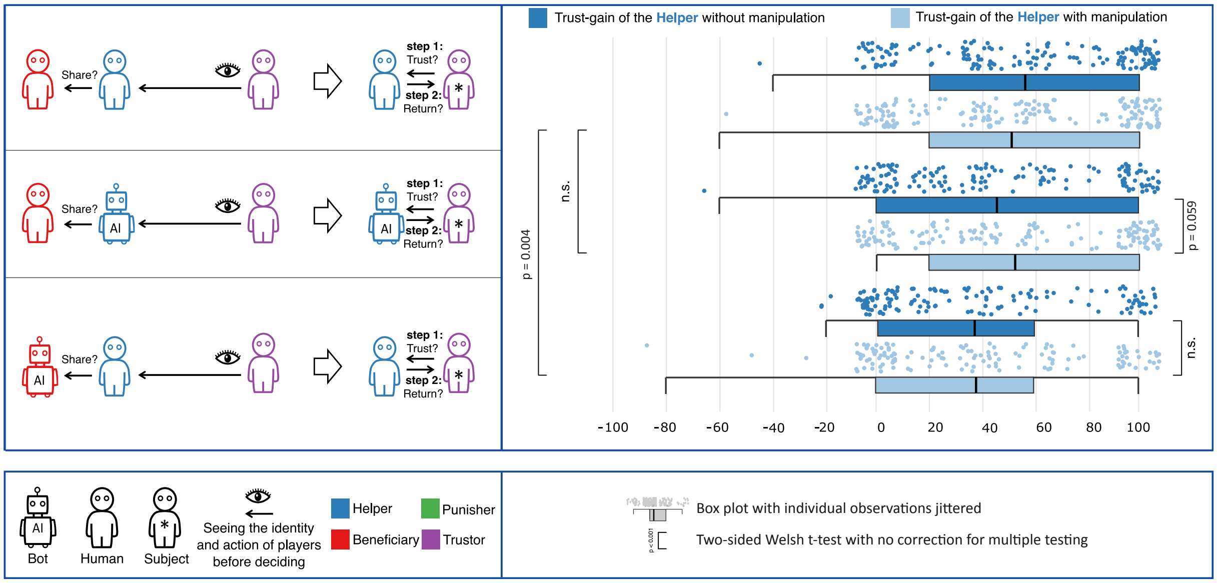

Supplementary Figure S6: **Results of Study 4 including participants who did not believe the experimental manipulation about norm consensus over Helpers' behavior in their condition.** The number of participants is 165, 152 and 141 for the pairs of plotted bars form top to bottom.

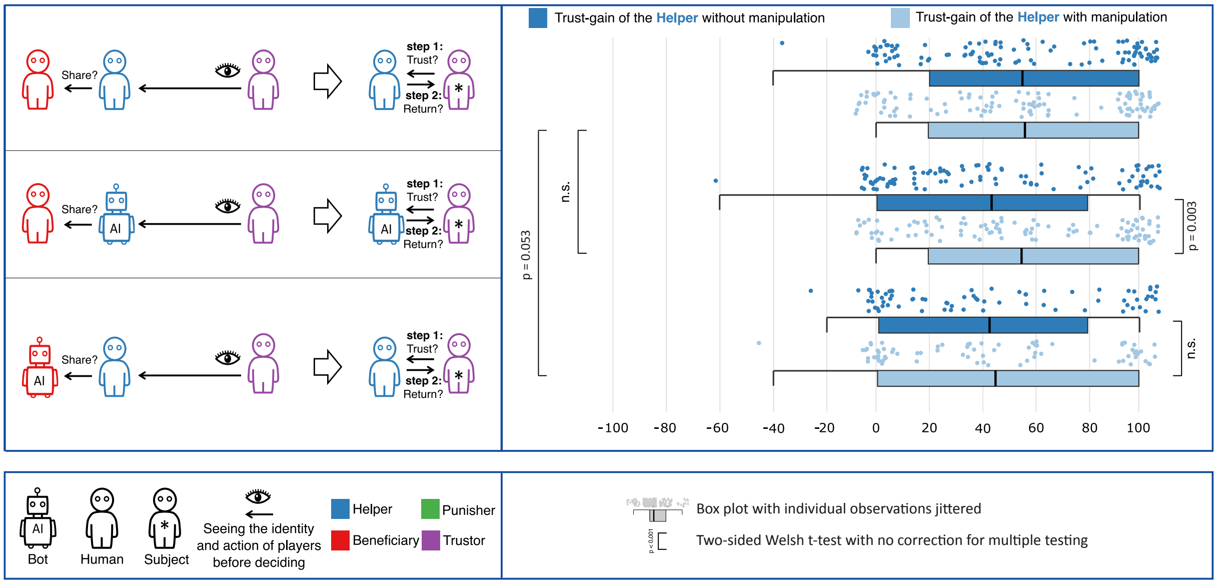

Supplementary Figure S7: **Results of Study 4 restricted to people who passed the manipulation check in both Study 2 and Study 4.** The number of participants is 111, 102 and 74 for the pairs of plotted bars from top to bottom.

Supplementary Note 3: Robustness Analysis of Study 5

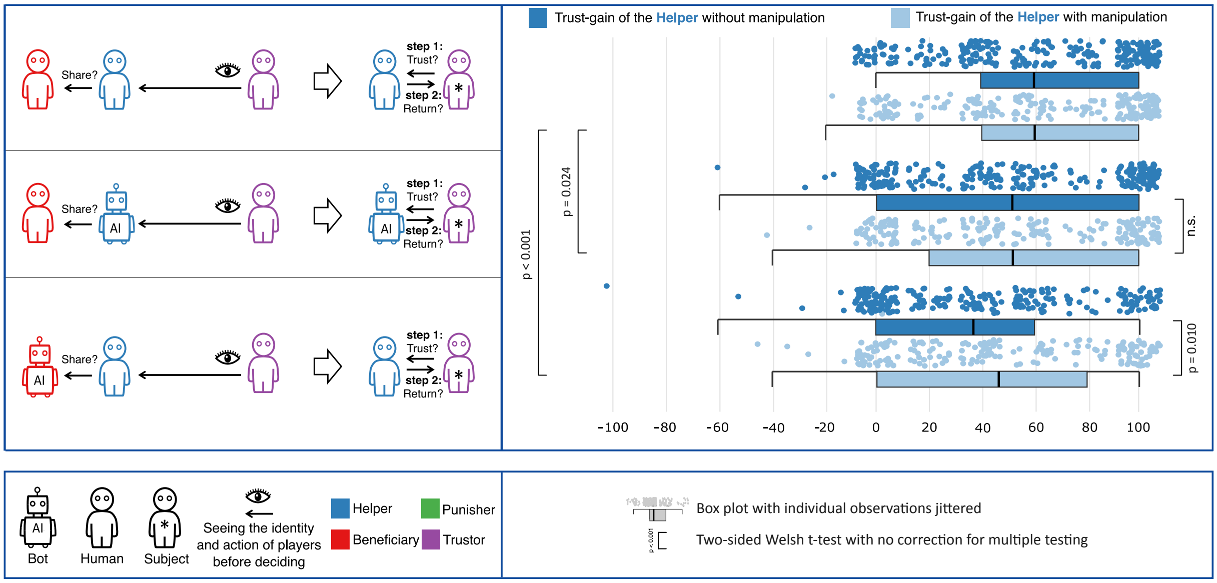

Supplementary Figure S8: **Results of Study 5 restricted to people who have correctly identified who were bots in the experimental condition.** The number of participants is 260, 234, 277, 214, 265 and 201 corresponding to the order of the plotted bars from top to bottom.

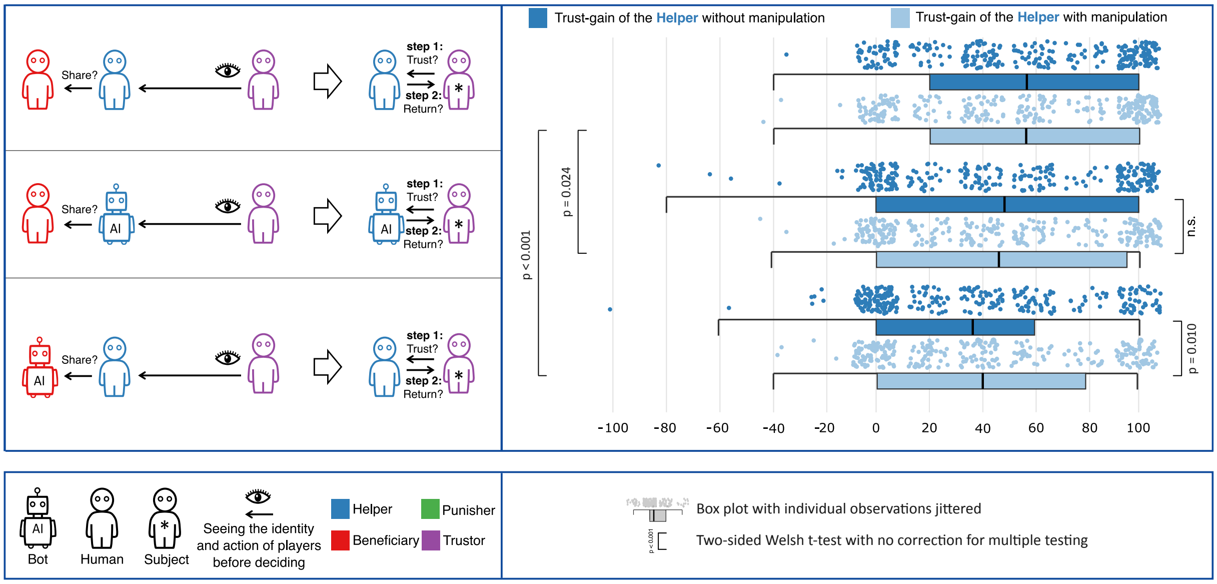

Supplementary Figure S9: **Results of Study 5 including participants who did not believe the experimental manipulation about the norm consensus over Helpers' behavior in their condition.**The number of participants is 303, 300, 300, 302, 300 and 300 corresponding to the order of the plotted bars from top to bottom.

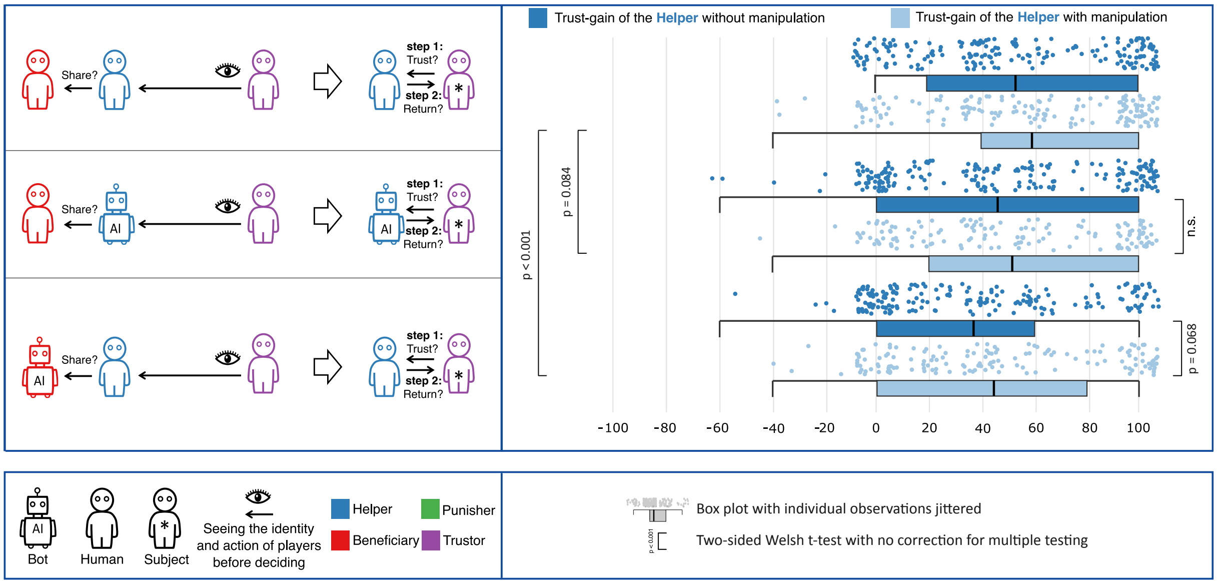

Supplementary Figure S10: **Results of Study 5 restricted to people who have never taken part in any of the previous studies.**The number of participants is 189, 163, 199, 144, 189 and 150 corresponding to the order of the plotted bars.

Supplementary Note 4: Complete Regression Tables for Study 3

Variables	Estimate	p-value
Intercept	18.771	$p = 0.003$
	(6.519, 31.023)	
**Consensus**	**0.257**	**p < 0.001**
	(0.133, 0.380)	
Norm	1.130	$p = 0.766$
	(-6.329, 8.589)	
Empirical Expectations	-0.012	$p = 0.855$
	(-0.137, 0.114)	
S-P1(B)-P2(P)-P3(P)	-2.442	$p = 0.421$
	(-8.391, 3.506)	
S-P1(P)-P2(B)-P3(P)	-10.082	$p = 0.002$
	(-16.504, -3.661)	
Age 30-50	5.413	$p = 0.060$
	(-0.239, 11.065)	
Age above 50	3.796	$p = 0.305$
	(-3.455, 11.047)	
Non-Hispanic White	4.608	$p = 0.090$
	-0.712, 9.929	
Income $30,000-$49,999	-2.830	$p = 0.463$
	(-10.394, 4.735)	
Income $50,000-$69,999	-1.175	$p = 0.769$
	(-9.019, 6.668)	
Income $70,000-$99,999	0.878	$p = 0.834$
	(-7.325, 9.080)	
Income $100,000 and more	5.913	$p = 0.166$
	(-2.465, 14.290)	
Income: prefer not to say	2.205	$p = 0.796$
	(-14.499, 18.910)	
College degree	-7.109	$p = 0.019$
	(-13.032, -1.186)	
More than college	-0.642	$p = 0.867$
	(-8.165, 6.881)	
Male	-1.056	$p = 0.669$
	(-5.899, 3.787)	
Midwest	-1.344	$p = 0.741$
	(-9.324, 6.637)	
South	-6.370	$p = 0.074$
	(-13.356, 0.617)	
West	-1.834	$p = 0.645$
	(-9.638, 5.971)	

Supplementary Table S5: **The relationship between the Trustor's beliefs of norm-consensus over the Helper's sharing behavior and the trust the Helper gains from sharing in Study 3.** Each experimental condition is denoted by "S" that stands for sharing, signaling that the Helper is matched with the Trustor in the trust game, furthermore the identity of participants in the third-party punishment game are signaled as, either a "P," a person, or a "B," a bot in parenthesis after the role they fulfill: P1 (Helper), P2 (Beneficiary), and P3 (Punisher), respectively.

Variables	Estimate	p-value
Intercept	-9.242	$p = 0.069$
	(-19.210, 0.74)	
**Consensus**	**0.109**	**p = 0.042**
	(0.004, 0.215)	
Norm	12.112	$p < 0.001$
	(7.047, 17.177)	
Empirical Expectations	-0.061	$p = 0.230$
	(-0.161, 0.039)	
P-P1(B)-P2(P)-P3(P)	4.955	$p = 0.087$
	(-0.727, 10.637)	
P-P1(P)-P2(B)-P3(P)	-8.324	$p = 0.005$
	(-14.118, -2.530)	
P-P1(P)-P2(P)-P3(B)	0.715	$p = 0.805$
	(-4.972, 6.402)	
Age 30-50	-2.833	$p = 0.241$
	(-7.575, 1.909)	
Age above 50	-11.514	$p < 0.001$
	(-17.549, -5.478)	
Non-Hispanic White	-2.285	$p = 0.318$
	(-6.770, 2.201)	
Income $30,000-$49,999	0.832	$p = 0.799$
	(-5.561, 7.225)	
Income $50,000-$69,999	2.316	$p = 0.490$
	(-4.261, 8.894)	
Income $70,000-$99,999	4.737	$p = 0.166$
	(-1.962, 11.436)	
Income $100,000 and more	5.589	$p = 0.109$
	(-1.248, 12.427)	
Income: prefer not to say	3.718	$p = 0.540$
	(-8.165, 15.601)	
College degree	1.572	$p = 0.518$
	(-3.200, 6.345)	
More than college	1.900	$p = 0.566$
	(-4.594, 8.394)	
Male	5.734	$p = 0.005$
	(1.690, 9.777)	
Midwest	1.611	$p = 0.626$
	(-4.877, 8.099)	
South	2.384	$p = 0.416$
	(-3.362, 8.131)	
West	4.942	$p = 0.128$
	(-1.423, 11.306)	

Supplementary Table S6: **The relationship between the Trustor's beliefs of norm-consensus over the Punisher's punishing behavior and the trust the Punisher gains from punishing in Study 3.** Each experimental condition is denoted by "P" that stands for punishment, signaling that the Punisher is matched with the Trustor in the trust game, furthermore the identity of participants in the third-party punishment game are signaled as, either a "P," a person, or a "B," a bot in parenthesis after the role they fulfill: P1 (Helper), P2 (Beneficiary), and P3 (Punisher), respectively.

Supplementary Note 5: Sample Composition of Study 1–5

N	**Study 1** 299	**Study 2** 3761	**Study 3** 2514	**Study 4** 473	**Study 5** 2077
**Age** (%)					
Below 30	0.20 (0.40)	0.29 (0.45)	0.29 (0.45)	0.22 (0.42)	0.18 (0.39)
30 to 50	0.66 (0.47)	0.55 (0.50)	0.53 (0.50)	0.55 (0.50)	0.57 (0.50)
Above 50	0.14 (0.34)	0.16 (0.37)	0.18 (0.39)	0.23 (0.42)	0.25 (0.43)
**Race** (%)					
White	0.79 (0.41)	0.79 (0.41)	0.74 (0.44)	0.81 (0.40)	0.80 (0.40)
American Indian or Alaska Native	0.01 (0.12)	0.01 (0.11)	0.01 (0.09)	0.01 (0.09)	0.01 (0.12)
Asian or Asian American	0.09 (0.29)	0.09 (0.29)	0.12 (0.32)	0.11 (0.31)	0.09 (0.29)
Black/African American	0.15 (0.36)	0.17 (0.56)	0.11 (0.31)	0.07 (0.25)	0.08 (0.28)
Hispanic/Latino(a)	0.07 (0.25)	0.06 (0.24)	0.07 (0.25)	0.05 (0.22)	0.05 (0.22)
Middle Eastern or North African	0.01 (0.12)	0.00 (0.05)	0.00 (0.07)	0.00 (0.07)	0.00 (0.06)
Other	0.01 (0.10)	0.01 (0.08)	0.01 (0.10)	0.01 (0.08)	0.01 (0.11)
**Income** (%)					
Less than $30,000	0.21 (0.41)	0.21 (0.41)	0.18 (0.38)	0.19 (0.39)	0.20 (0.40)
$30,000-$49,999	0.23 (0.42)	0.23 (0.42)	0.22 (0.42)	0.23 (0.42)	0.21 (0.41)
$50,000-$69,000	0.20 (0.40)	0.19 (0.40)	0.21 (0.40)	0.16 (0.37)	0.19 (0.39)
$70,000-$99,000	0.17 (0.37)	0.18 (0.39)	0.18 (0.39)	0.19 (0.39)	0.19 (0.39)
$100,000 and above	0.17 (0.37)	0.16 (0.37)	0.18 (0.39)	0.20 (0.40)	0.18 (0.39)
Prefer not to say	0.01 (0.12)	0.03 (0.16)	0.03 (0.17)	0.03 (0.17)	0.03 (0.16)
**Education** (%)					
Less than college	0.34 (0.47)	0.34 (0.47)	0.27 (0.44)	0.30 (0.46)	0.31 (0.46)
College degree (2 or 4 years)	0.54 (0.50)	0.51 (0.50)	0.55 (0.50)	0.56 (0.50)	0.52 (0.50)
More than college	0.12 (0.33)	0.15 (0.36)	0.18 (0.39)	0.14 (0.34)	0.17 (0.38)
**Gender** (%)					
Male	0.59 (0.49)	0.48 (0.50)	0.50 (0.50)	0.45 (0.50)	0.47 (0.50)
Female	0.41 (0.49)	0.51 (0.50)	0.49 (0.50)	0.53 (0.50)	0.52 (0.50)
Other	0.01 (0.08)	0.00 (0.07)	0.01 (0.08)	0.01 (0.08)	0.01 (0.08)
**Region** (%)					
Northeast	0.20 (0.40)	0.18 (0.39)	0.17 (0.38)	0.16 (0.37)	0.18 (0.39)
Midwest	0.21 (0.41)	0.22 (0.42)	0.20 (0.40)	0.21 (0.41)	0.23 (0.42)
South	0.41 (0.49)	0.39 (0.49)	0.40 (0.49)	0.41 (0.49)	0.38 (0.48)
West	0.17 (0.38)	0.20 (0.40)	0.22 (0.42)	0.22 (0.41)	0.21 (0.41)
**Comprehension** (mean % correct (SD))	1.00 (0.00)	0.98 (0.05)	0.83 (0.21)	0.95 (0.09)	0.98 (0.05)
**Compensation** (mean (SD) in $)	2.00 (0.00)	1.79 (0.78)	2.95 (0.53)	4.97 (6.20)	1.22 (0.30)

Supplementary Table S7: **Sample composition of Studies 1–5.**

Supplementary Note 6: Comparing Samples Across Studies 2–5

	Study 2	Study 3	SMD
N	3761	2066	
**Age** (%)			
Below 30	0.29 (0.45)	0.30 (0.46)	0.026
30 to 50	0.55 (0.50)	0.51 (0.50)	0.070
Above 50	0.16 (0.37)	0.19 (0.39)	0.061
**Race** (%)			
White	0.79 (0.41)	0.76 (0.43)	0.085
American Indian or Alaska Native	0.01 (0.11)	0.01 (0.09)	0.047
Asian or Asian American	0.09 (0.29)	0.12 (0.33)	0.106
Black/African American	0.17 (0.56)	0.08 (0.27)	0.211
Hispanic/Latino(a)	0.06 (0.24)	0.07 (0.26)	0.054
Middle Eastern or North African	0.00 (0.05)	0.00 (0.06)	0.008
Other (mean (SD))	0.01 (0.08)	0.01 (0.10)	0.032
**Income** (%)			
Less than $30,000	0.21 (0.41)	0.17 (0.38)	0.096
$30,000-$49,999	0.23 (0.42)	0.21 (0.41)	0.037
$50,000-$69,000	0.19 (0.40)	0.20 (0.40)	0.011
$70,000-$99,000	0.18 (0.39)	0.19 (0.39)	0.014
$100,000 and above	0.16 (0.37)	0.20 (0.40)	0.109
Prefer not to say	0.03 (0.16)	0.03 (0.17)	0.009
**Education** (%)			
Less than college	0.34 (0.47)	0.28 (0.45)	0.127
College degree (2 or 4 years)	0.51 (0.50)	0.53 (0.50)	0.036
More than college	0.15 (0.36)	0.19 (0.39)	0.108
**Gender** (%)			
Male	0.48 (0.50)	0.49 (0.50)	0.015
Female	0.51 (0.50)	0.50 (0.50)	0.024
Other	0.00 (0.07)	0.01 (0.09)	0.056
**Region** (%)			
Northeast	0.18 (0.39)	0.18 (0.38)	0.015
Midwest	0.22 (0.42)	0.21 (0.41)	0.024
South	0.39 (0.49)	0.39 (0.49)	0.002
West	0.20 (0.40)	0.22 (0.41)	0.037

Supplementary Table S8: **Comparing the demographic composition of Study 2 and Study 3**. The sample of Study 3 is restricted to participants who showed the same level of comprehension as those in Study 2, i.e., answered at least three of four comprehension check questions correctly in both sets. SMD stands for standardized mean difference.

	Study 2	Study 4	SMD
**N**	939	458	
**Age** (%)			
Below 30	0.29 (0.45)	0.22 (0.42)	0.141
30 to 50	0.55 (0.50)	0.55 (0.50)	0.010
Above 50	0.16 (0.37)	0.23 (0.42)	0.168
**Race** (%)			
White	0.78 (0.41)	0.81 (0.40)	0.053
American Indian or Alaska Native	0.01 (0.11)	0.01 (0.09)	0.039
Asian or Asian American	0.10 (0.30)	0.11 (0.31)	0.030
Black/African American	0.17 (0.56)	0.07 (0.25)	0.244
Hispanic/Latino(a)	0.06 (0.23)	0.05 (0.22)	0.022
Middle Eastern or North African	0.00 (0.06)	0.00 (0.07)	0.019
Other	0.01 (0.07)	0.01 (0.08)	0.016
**Income** (%)			
Less than $30,000	0.22 (0.42)	0.19 (0.39)	0.074
$30,000-$49,999	0.24 (0.43)	0.23 (0.42)	0.022
$50,000-$69,000	0.18 (0.39)	0.16 (0.37)	0.065
$70,000-$99,000	0.18 (0.38)	0.19 (0.39)	0.037
$100,000 and above	0.15 (0.36)	0.20 (0.40)	0.114
Prefer not to say	0.02 (0.15)	0.03 (0.17)	0.038
**Education** (%)			
Less than college	0.33 (0.47)	0.30 (0.46)	0.051
College degree (2 or 4 years)	0.53 (0.50)	0.56 (0.50)	0.052
More than college	0.14 (0.35)	0.14 (0.34)	0.006
**Gender** (%)			
Male	0.48 (0.50)	0.47 (0.50)	0.020
Female	0.52 (0.50)	0.53 (0.50)	0.017
Other	0.01 (0.07)	0.01 (0.08)	0.016
**Region** (%)			
Northeast	0.17 (0.38)	0.16 (0.37)	0.023
Midwest	0.22 (0.42)	0.21 (0.41)	0.030
South	0.38 (0.49)	0.41 (0.49)	0.048
West	0.22 (0.41)	0.22 (0.41)	0.006

Supplementary Table S9: **Comparing the demographic composition of Study 2 and Study 4**. SMD stands for standardized mean difference.

Supplementary Note 7: Contrasting Study 4 and Study 5

The overarching goal of both Study 4 and 5 is the same. Specifically, to investigate if manipulating participants' beliefs about the consensus over Helpers' behaviors in their specific experimental condition induces a distinction between the trust placed in norm-followers and non-followers. While the goals of these studies are similar, their research designs are different which make the strengths and weaknesses of each study complementary.

Study 4 uses the same participants as Study 2, therefore, participants' Study 2 decisions act as controls for their Study 4 decisions. The research question in this repeated measures design highlights the change within a person, relative to the variability of change scores. On the other hand, in Study 5 we collect data from a novel sample (65% have not taken part in any of the previous data collections). When independent groups are analyzed, the research question focuses on the difference between groups relative to the variability within groups.

As Morris and DeShon note, alternate experimental designs control for different sources of bias (*1*), which they summarize in their Table 1. The within-person design (Study 4) is most susceptible to time-effects, such as maturation (natural change in subjects' views over the trust placed in norm-followers, non-followers or both which impact the trust-gain between the two time-points when participants are observed) or history (learning from previous experiences which could impact the trust-gain). The between-person design is most susceptible to selection effects (minimized by random assignment, but may be subject to differential attrition which is specifically addressed in Supplementary Note 13) and differential time-effects (these are also minimized as a result of random assignment as treatment and control groups were measured at the same time).

To address some of the shortcomings of the within-person design of Study 4, we performed the following robustness check. We include a question in the experiment that asks about how

the two studies (Studies 2 and 4) compare (specifically by asking: "*You have been invited to this study as a result of your participation in a study earlier. How do you think these two studies compare?*") with options presented on a 4-point scale ranging from the studies being identical to being completely different, and with a fifth option of "*I don't know as I do not remember the details of the previous study.*" In order to alleviate concerns about recall, we carry out our analysis only on participants who disclosed they did not remember the study (N = 172, 59.93% of the sample), and those who believed that the two studies were different (N = 21, 7.31% of the sample). In sum, we drop participants who believed the studies were identical (which technically they were not, but they only differed in a single sentence, N = 10, 3.48% of the sample), and those who thought the studies were similar (N = 84, 29.27% of the sample). We re-estimate the comparisons reported in Figure 4 in the main paper, on the smaller sample described above, and present these in Figure S11, which offer similar substantive conclusions.

Last but not least, we investigate the biases specific to Study 5, which center on selective attrition. We group these results together with similar investigations in the other studies and discuss them in Supplementary Note 13.

Meta Analysis: Pooling the Estimates of Studies 4 and 5

To bolster our quest for robustness, we also conduct a meta-analysis of Studies 4 and 5. Doing so, we follow closely Morris and DeShon who discuss how effect size estimates may be combined across different studies that employ different designs (*1*). They suggest that: "*meta-analysis on effect sizes from alternate designs can be performed using standard procedures, as long as (a) the effect sizes are first transformed into a common metric and (b) the appropriate sampling variance formulas are used when estimating the mean and testing for homogeneity of effect size*" (*ibid*, p.119). We undertake these steps consecutively.

First, we transform the effect sizes into a common metric on the basis of equations (11) and

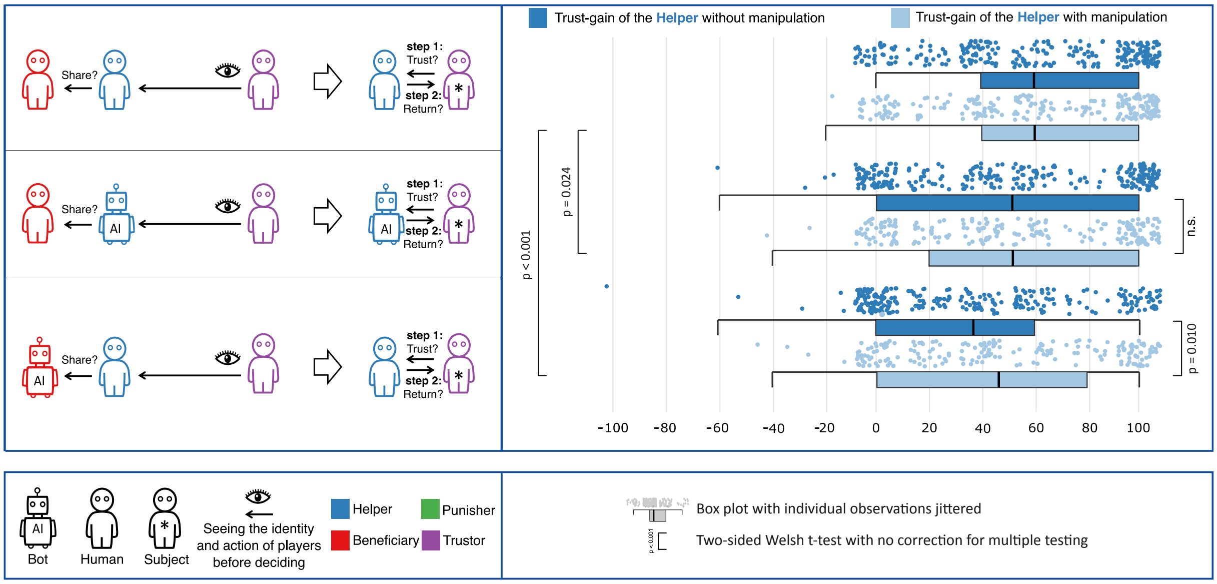

Supplementary Figure S11: **Results of Study 4 restricted to people who believed the norm manipulation and had no/erroneous recall of Study 2.** The number of unique participants is 72, 71 and 50 corresponding to the plotted order of pairs of bars.

(12) from (*1*) which give the following cross-walk:

$$d_{IG} = d_{RM}\sqrt{2(1-\rho)}, \tag{1}$$

leading to an estimate for the effect size based on the estimate of change scores d_{RM}, expressed in raw score change, where ρ is the correlation between pre- and post-test scores. In our case, ρ is the correlation between the trust-gain with and without the norm-manipulation in Study 4, following Morris and DeShon who state that "*an aggregate of the correlational data across the single-group pretest–posttest designs provides the best estimate of the population correlation*" (*ibid*, p.120).

Second, we compute the sampling variances defined in Table 2 in (*1*) for the two studies separately.

The sampling variance for Study 4 is:

$$\mathrm{Var}_{RM} = \left(\frac{1}{n}\right)\left(\frac{n-1}{n-3}\right)(1 + n\delta_{RM}^2) - \frac{\delta_{RM}^2}{[c(n-1)]^2}, \tag{2}$$

where n is the number of paired observations, δ_{RM} is the population effect size in the change score metric, specifically $\delta_{RM} = \frac{\mu_{D,E}}{\sigma_{D,E}}$ and $\sigma_{D,E}$ is the standard deviation of the change scores, and $\mu_{D,E}$ is the mean of the change scores. Additionally, $c(.)$ is the bias function, where $c(df) = 1 - \frac{3}{4df-1}$, and $df = n - 1$, where n continues to refer to the number of paired observations.

The sampling variance for Study 5 is:

$$\mathrm{Var}_{IG} = \left(\frac{1}{\tilde{n}}\right)\left(\frac{N-2}{N-4}\right)(1 + \tilde{n}\delta_{IG}^2) - \frac{\delta_{IG}^2}{[c(N-2)]^2}, \tag{3}$$

where $\tilde{n} = n_E n_C/(n_E + n_C)$ and by n_E the size of the experimental group is meant, in our case those who received information about the norm consensus, and n_C the size of the experimental group who did not receive the norm-consensus information; and N is the combined number of observations, $n_E + n_C$. Additionally, $c(.)$ is the bias function, where $c(df) = 1 - \frac{3}{4df-1}$, and $df = n_E + n_C - 2$. Note that the population effect size is replaced by its estimate when calculating the sampling variance, and the estimate is taken to be the simple (unweighted) average of the effect sizes across the two studies.

Now that we have the effect sizes expressed in the same metric, we may combine these by weighting the estimates from the individual studies by the reciprocal of the sampling variance to provide the most accurate estimates (the summations include only two terms for Studies 4 and 5):

$$\bar{\delta} = \frac{\sum_{i=1}^{2} w_i d_i}{\sum_i^2 w_i}, \tag{4}$$

where w_i are given above and are the reciprocal of the sampling variance: $1/\mathrm{Var}_{RM}$ and $1/\mathrm{Var}_{IG}$, respectively for the two studies. Note that the population effect size is replaced by its estimate when calculating the sampling variance, and the estimate is taken to be the simple (unweighted) average of the effect sizes across the two studies.

Study	Design	n_T	n_C	d_{IG}	d_{RM}	c	Var_{IG}	w_{IG}	$w_{IG}d_{IG}$	Var_{RM}	w_{RM}	$w_{RM}d_{RM}$
Study 4	RM	102	102	0.31	0.30	0.99	0.01	91.31	28.00	0.01	119.46	36.43
Study 5	IG	231	295	0.02	0.02	1.00	0.01	126.52	2.15	0.01	127.99	2.16

Supplementary Table S10: **The trust gain of the bot Helper as a result of the norm-consensus manipulation**. n_T stands for the size of the group that received the norm-manipulation, and n_C stands for the size of the group that did not receive the norm-manipulation; d is the effect size estimate; c is the value of the bias function; Var_{IG} and Var_{RM} are the sampling variances computed on the basis of equations (2) and (3) and the ws stand for the respective weights used to weight the effect size estimates.

Study	Design	n_T	n_C	d_{IG}	d_{RM}	c	Var_{IG}	w_{IG}	$w_{IG}d_{IG}$	Var_{RM}	w_{RM}	$w_{RM}d_{RM}$
Study 4	RM	74	74	0.07	0.06	0.98	0.02	58.29	3.90	0.01	82.71	5.13
Study 5	IG	221	293	0.21	0.19	1.00	0.01	123.39	25.66	0.01	143.34	27.64

Supplementary Table S11: **The trust gain of the Helper sharing with a bot Beneficiary as a result of the norm-consensus manipulation**. n_T stands for the size of the group that received the norm-manipulation, and n_C stands for the size of the group that did not receive the norm-manipulation; d is the effect size estimate; c is the value of the bias function; Var_{IG} and Var_{RM} are the sampling variances computed on the basis of equations (2) and (3) and the ws stand for the respective weights used to weight the effect size estimates.

For the test of homogeneity, we obtain the observed variance (the numerator is 2, since we are focusing on two studies):

$$\hat{\sigma}_d^2 = \frac{\sum_{i=1}^2 w_i (d_i - \bar{d})^2}{\sum_i^2 w_i}, \tag{5}$$

where w_i are given above and are the reciprocal of the sampling variance: $1/\text{Var}_{RM}$ and $1/\text{Var}_{IG}$, respectively for the two studies.

We also obtain the variance due to sampling error, which is estimated from the weighted average of the individual study variances:

$$\hat{\sigma}_e^2 = \frac{2}{\sum_{i=1}^2 \frac{1}{\hat{\sigma}_{e_i}^2}}, \tag{6}$$

where $\hat{\sigma}_{e_i}^2$ is the sampling variance as defined in (2) and (3). The effect size is viewed as homogeneous when $Q = k\hat{\sigma}_d^2/\hat{\sigma}_e^2$ is checked against a χ^2 distribution with $k - 1$ degrees of freedom, where k is the number of studies, in this case, 2, and the null-hypothesis cannot be

rejected. Alternatively, Smith and Hunter suggest to calculate the ratio $\hat{\sigma}_d^2/\hat{\sigma}_e^2 > 0.75$, and combine estimates, i.e., accept them as homogeneous, when the 75% rule is met.

We produce a comparable table to Table 3 in (*1*), separately for both of the experimental conditions determined by the identity of players in Stage 1, and present all the metrics in Tables S10–S11 for the sake of completeness. From Table S10 we calculate the effect size $d_{IG} = 0.138$ and $d_{RM} = 0.156$, and the corresponding variances $\hat{\sigma}_{IGd}^2 = 0.01$ and $\hat{\sigma}_{RMd}^2 = 0.01$, respectively. The test for homogeneity yields p-values of $p = 0.323$ and $p = 0.312$, respectively. Therefore, while there is visible variation, the null-hypothesis of homogeneity cannot be rejected in this case, which suggests that these estimates could be combined. The 75% rule in contrast in this case suggests otherwise. From here, the 90% confidence intervals are $[-0.098, 0.375]_{IG}$ and $[-0.075, 0.387]_{RM}$ (*2*). These intervals contain zero, which means that *if* the estimates are combined the effect is assessed with uncertainty, and while large effects are possible, a null-effect may not be ruled out.

From Table S11 we calculate the effect size $d_{IG} = 0.163$ and $d_{RM} = 0.145$, and the corresponding $\hat{\sigma}_{IGd}^2 = 0.004$ and $\hat{\sigma}_{RMd}^2 = 0.004$, respectively. The test for homogeneity yields p-values of $p = 0.013$ and $p = 0.024$, respectively. Therefore, we may reject the null hypothesis in this case, which warrants against combining these estimates and suggests that these quantities (which are theoretically different), may actually be different. While keeping this in mind, the 90% confidence intervals are $[0.055, 0.271]_{IG}$ and $[0.042, 0.248]_{RM}$, and the 95% confidence intervals do not contain zero either (*2*) which means that *if* the estimates are combined the data are consistent with a positive effect of norm-information on the trust-gain.

Taken all together, Studies 4 and 5 are complementary, each with complementary evidence that clarifying the norm that applies would impact the behavior of Trustors. However, there is relatively strong evidence that in this case the estimands are theoretically different quantities given our results for the test of homogeneity. Therefore, the scope conditions for the circum-

stances under which norm-signals may be more or less effective shall be the subject of future work.

Supplementary Note 8: Justifications for Helping and Trust Decisions

In Studies 2, 4 and 5 participants were asked to elaborate on the reasons why they made the decision(s) they have. The most central to our argument are responses given by Helpers and Trustors. Specifically, Helpers were asked "*We would like to know how you made your decision about whether to share with Player 2. In a few sentences, please write your response in the box below.*" Trustors were asked "*We would like to know how you made your decision about how much money to send to Player 1. In a few sentences, please write your response in the box below.*" Note that to avoid leading participants in any way in the experimental setup we simply referred to participants as players, rather than Beneficiaries, Helpers, Punishers and Trustors; therefore Player 2 in the context of our paper is the Beneficiary, while Player 1 is the Helper.

Responses were first coded to establish a concise set of themes. After developing this coding scheme, two independent coders coded all responses (one pair coded Studies 2 and 5, while another pair coded Study 5), then compared their codes, discussed and adjudicated between their answers, agreeing on unified codes which we analyze. The independent coders were research assistants who were not familiar with the hypotheses tested in the study and how these data would be deployed to avoid any conscious or unconscious biases.

Participants were not aware that we would ask them to justify their decisions when they have made them. This approach alleviates concerns that participants would avoid decisions, such as not sharing their resources with a person, that they deem inappropriate or would make them feel judged, but that they would in fact engage in in short of such pressures. While for these reasons we believe decisions themselves do not carry such biases, justifications might simply be a result of motivated reasoning. For example, participants may have simply wanted to maximize their bonus and act selfishly, but they might have justified their selfish decision by highlighting that

they were in dire need of the money and that they were short on bills. These pressures apply to all experimental conditions; while it is possible that the severity of these pressures vary by the identity of the players signaled to participants: participants might have been less likely to hide selfish reasons when Beneficiaries were bots, for example.

Helpers' Justifications

To classify Helpers' justifications, the following 12 codes were developed and applied for all responses. Codes are not exclusive, as many justifications (45.7%) contain more than one reason, on average 1.59 reasons (sd = 0.75). The average justification contains 26.0 words (sd = 16.12), with a range of 1 to 102. Note that some codes, like **1**, have multiple sub-codes. Therefore, a response could have been coded as **1**, **1a**, **1b** or **1c**. Note further that participants were aware of the possibility that some players may be bots—even if their specific experimental conditions did not have any bots. For this reason they may have referenced their thinking about bots, and sometimes gave complete reasons how their decisions might have differed if, e.g., the Beneficiary they were matched with was a bot, and not a person. Below are all codes:

1 The decision was based on what the Punisher might do.

- **1a** I wanted to avoid punishment.
- **1b** I chose to take/avoid a risk: I may/may not get punished.
- **1c** Referencing the way in which Punishers make their decision.

2 The decision was made on the basis of the identity of the Beneficiary (either because they were a bot or because they were a person).

- **2a** Given who the Beneficiary is, they may need/not need the money.
- **2b** Given who the Beneficiary is, their feelings could be/could not be hurt.

3 The decision was made to impress the Trustor.

3a Specifically, to have the Trustor to think of the Helper as a nice/fair/trustworthy person.

3b Specifically, to ensure that the Trustor sends more of their resources to the Helper.

4 The decision was made on the basis of some higher-level or universal principle. E.g., to ensure "equality" or because the participant is a fair/moral/ethical person and/or their actions were a fair/generous/moral/ethical thing to do.

5 The decision was made on the basis of the identity (either universal or temporal characteristic) of the Helper as a "nice person" or a "person in need."

6 The decision was made to maximize the Helper's monetary gain without giving an indication if it is in reference to stage 1, stage 2, or the combination.

7 The decision was made on the basis of the reciprocity principle: the Helper treated the Beneficiary in such a way they would expect to be treated in their shoes.

8 The decision was made with the owner of the bot in mind.

9 The participant was confused (most often did not believe the identity of players signaled).

9a Based on the Helper's justification, the participant misunderstood the rules of the game in some way. E.g., they indicated that the money they would send to Beneficiary would be doubled.

9b Based on the Helper's justification, the participant misunderstood who has information about the identities of the players, which was explicitly signaled to all decision makers.

10 The reason is rooted in what all players should do.

11 The decision was made because the Trustor cannot be influenced.

12 The justification did not meet any of the above classifications.

In Table S12 we provide a typical response for each code; and Table S13 provides the distribution of justifications across experimental conditions. We find support for the assertions that Helpers consider Trustors' decisions when they decide whether or not to share their resources with the Beneficiaries. These strategic considerations are more prevalent when Beneficiaries and Punishers are people (23.8%), compared to when Beneficiaries are bots (19.7%). In fact, impressing the Trustor to send more money was the second most common reason mentioned in the human-only condition, while in the condition when the Beneficiary was a bot this was only the sixth most common. In substantive terms, Helpers are highly concerned about what their behavior might *mean* to Trustors when they interact with other humans, but these considerations do not disappear, as they do try to ponder over the meaning of their actions in the eyes of others, even when they interact with bots. While this aspect of signaling has been the main focus of our argument, helping norms (which are implied by Helpers' concerns about the punishment they might receive), also figured into Helpers' decision making. Specifically, Helpers wanted to avoid punishment when they were paired with human Beneficiaries (9.8%), while they reasoned this way as well when they were paired with a bot Beneficiary (10.8%). References to higher-level principles were made much more frequently when Beneficiaries were people (47.0%) compared to when Beneficiaries were bots (21.0%). Many participants stated simply that they made their decision *because* the Beneficiary was a bot without giving much further detail and that bots did not need money. These considerations clearly crowd out, but do not eliminate, concerns about punishment or the desire to impress Trustors, or thoughts of higher-level principles, such as fairness. In sum, signaling motives and higher-level principles/ethics

dominate Helpers' justifications, albeit with variation by condition.

Code	Example answer
**1**	*I could keep all of my money depending on the choices of player 3*
**1a**	*I did not want to be punished by player 3 for not sharing.*
**1b**	*I will take the risk of player three punishing me if it means a chance at keeping the money. If they don't punish me, then I keep all the money, but if they do, i would end up with 50 cents which is the same as if i would have shared with player 2.*
**1c**	*I decided to keep the money because I don't know what Player 3 will do.*
**2**	*I decided not to share with Player 2 because it was a bot.* Or: *I think I should share even though the player is a robot.* I.e., referencing the identity of the Beneficiary led to both outcomes.
**2a**	*I didn't share with Player 2 because they were a bot, and they wouldn't really benefit at all by my sharing.*
**2b**	*I felt that the bot had no feelings so it wasn't amoral to keep the money.*
**3**	*I wanted it to reflect positively on the next stages.*
**3a**	*I would hope player 4 would think I was generous and would return half the money to him or her.*
**3b**	*I am willing to share to better my chances of a better share of the pot in the next stage.*
**4**	*It is the right thing to do.* Or: *I like to be equal and share the wealth. There is no reason I should not share the money.*
**5**	*I'M IN THIS FOR MY OWN MONEY.* Emphasis original. Or: *I'm a nice person.* I.e., participants volunteered explanations that point to them being selfish/greedy/self-interested as well as being nice/fair/kind.
**6**	*My decision was based on the goal to maximize my payout.*
**7**	*I believe that most participants would make the same decision as me, if they were in my place.*
**8**	*At first I thought it would be futile to share with a bot because no one would really benefit but then I figured someone must own the bot and so I would be sharing with them.*
**9**	*I am also assuming these are bots and not actual people.*
**9a**	*I hope player two sends me back .75 cents.* The Beneficiary made no decisions in the game, therefore, they had no opportunity to send any money back.
**9b**	*So while player 3 probably wouldn't punish me for not sending to a bot, player 4 doesn't know it's a bot and might just think I'm selfish. Then again, maybe I remember wrong and player 4 knows player 2 was a bot. but I think since player 4 only joins later they aren't informed.*
**10**	*if we can all help each other out I think it'd be advantageous to all of us.*
**11**	*I didn't share with Player 2 because I think that Player 4 won't care either way if I shared or not and will probably keep their bonus to themselves anyway.*
**12**	*I decided how much money I wanted in to end.*

Supplementary Table S12: **Typical answers of Helpers matching each code introduced.** Note that in the experiment the language of Beneficiary (Player 2), Helper (Player 1), Punisher (Player 3) and Trustor (Player 4) were avoided. E.g., Helpers may have shared more if Players 2 were called "Beneficiary" as it suggest that they "need" to benefit etc.

Justification (%)	No bot	Bot Beneficiary
**1:** What the Punisher might do.	2.5	5.1
**1a:** Avoid punishment.	21.3	18.8
**1b:** Take/avoid risk.	1.9	2.9
**1c:** The way the Punisher makes decisions.	3.2	8.0
**2:** The identity of the Beneficiary.	5.7	26.1
**2a:** The Beneficiary's need for money.	1.6	16.9
**2b:** The Beneficiary's feelings.	0.0	0.6
**3:** Impress the Trustor.	7.0	9.6
**3a:** Impress the Trustor about the Helper's character.	7.0	5.1
**3b:** Impress the Trustor to send more.	27.3	18.2
**4:** Morality/ethics etc.	47.0	21.0
**5:** The character of the Helper.	9.8	4.5
**6:** Maximize gain.	9.2	7.0
**7:** Reciprocity principle.	1.6	3.8
**8:** Considering the owner of the bot.	3.2	2.5
**9:** Confused Helper.	0.0	0.3
**9a:** Misunderstood rules.	0.6	1.3
**9b:** Misunderstood who has what information.	0.6	1.0
**10:** What all players should do.	6.0	3.2
**11:** The Trustor cannot be influenced.	3.2	3.2
**12:** None of the above.	1.6	1.3

Supplementary Table S13: **The distribution of reasons for making a decision by the Helper.** The first column contains the reasons, the second the share of times that a specific reason was mentioned in the human-only condition, the third the share of times that a specific reason was mentioned in the condition when the Beneficiary was a bot.

Trustors' Justifications

To classify Trustors' justifications, 13 codes were developed and applied for all responses. Since our study used the strategy method, justifications were also categorized to reflect that some participants reasoned through their decisions when Helpers shared (A); when Helpers did not share (B); both when Helpers shared and when they did not (C); and in some cases based on the response it was unclear which of these Trustors justified (D). Which decision (sharing vs. not) was referred to showed some variation across experimental condition, but no consistent patterns emerged across studies.

Codes are not exclusive as many justifications contain more than one reason (33.5% in Study 2, 39.5% in Study 4, and 37.0% in Study 5; on average 1.39 (sd = 0.61), 1.47 (sd = 0.62), and 1.45 (sd = 0.66), respectively). The average justification contains 38.67 (sd = 25.74), 42.64 (sd = 22.43), and 41.37 (sd = 25.14) words with a ranges of 1 to 242, 329 and 201, across Studies 1, 3 and 4. Note that some codes, like **5**, have multiple sub-codes. Therefore, a response could have been coded as **5** (without further details), **5a**, **5b**, **5c** or **5d**. Note further that participants were aware of the possibility that some players may be bots—even if their specific experimental conditions did not have any bots; for this reason they may have referenced their thinking about bots, and sometimes gave complete reasons how their decisions might have differed, if the Helper/Beneficiary/Punisher were bots.

1 The decision rested on the principle of "consistency" of behavior, expressing what Helpers did in Stage 1 (helping/not helping), they will do in Stage 2 (sending money back/not sending money back).

2 The reasoning is rooted in considering the risk involved (taking/not taking a risk).

3 The decision is rested in the identity of the Trustor: being a fair/moral/ethical person and or regarding the action of sending/not sending money as fair/moral/ethical act. I.e., referencing some higher or universal principle, e.g., equality, or some situational element: the Trustor is a person in need of money.

4 The decision was motivated by wanting to reward/punish the Helper.

5 The decision was made on the basis of the identity of the Helper (either because they were a bot/or because they were a person).

5a The Helper needs/does not need money/makes no sense to give money to them.

5b The Helper does/does not have feelings which could be hurt, or the Helper has/does not have the capacity to empathize.

5c The Helper makes random decisions.

5d It is unclear how the Helper makes decisions.

6 The Helper needed the money/the Trustor wanted to help them.

7 The decision was based on a quality/characteristic the Trustor assumed the Helper had: they can be trusted/can not be trusted, are generous/are stingy, nice/are not nice, kind/are not kind, fair/unfair, etc.

8 The Trustor aimed to maximize their bonus (without further referencing any of the other justifications, e.g., hoping the Helper would share their resources without referencing that they would be consistent, or any other reason).

9 The decision was made based on the Helper's behavior without giving any further details (i.e., the Trustor differentiated, but did not explain why, simply restating what the decision was, not why it was made).

10 The decision was rooted in the Beneficiary's identity.

11 The participant was confused.

11a The justification gave an indication that the Trustor misunderstood the rules of the game in some way. E.g., assumed that non-sharing Helpers are always punished; or assumed that they did not know how the Helper decided earlier (which was explicitly signaled to them).

11b The justification gave an indication that the Trustor misunderstood who has information about the identities of the players, which was explicitly signaled to all decision

makers.

11c The Trustor did not believe the identity of whom they were paired with (bot/person).

12 The decision was made so that the Helper would "do the right thing" in the future.

13 The justification did not meet any of the above classifications.

In Table S14 we provide some typical responses for each code. Followed by this, we make several comparisons. Specifically, we start by outlining how the distribution of justifications in Study 2 differed across experimental conditions (Table S15). We find strong support for the assertion that Trustors considered Helpers' decisions, and reflected mostly on the consistency they anticipated of the Helpers' actions across Stage 1 and Stage 2, which varied with experimental condition. Importantly, in the human-only condition 41.6% referred to this principle, and while this reasoning was still the most prominent one, participants referred to consistency of the bot Helper only in 31.3% of cases and intuited such consistency of Helpers 27.9% of the time when they were paired with bot Beneficiaries. In sum, the strength of the signal about Helpers diminishes considerably when they were paired with bot Beneficiaries in the eyes of Trustors.' These justifications also allude to norms, and while (understandably) Trustors do not use the language employed in the scholarly literature, a few participants expressed that they wished to reward or punish Helpers. These terms reference norm enforcement, at twice the rate in the human-only condition (8.3%) over the condition when the Helper is a bot (4.3%), with the condition where the Beneficiary is a bot in the middle (6%). Not surprisingly, the Helper's identity figures into Trustors' justifications at much higher rates when the Helper is a bot (12.3%) compared to when they are a person (0.6% and 1.7%, respectively), and most of the Trustors' concerns focus on the way in which Helpers make decisions (asserting that they make them randomly in 2.1% of the time, and expressing that they do not know how they make them/how the bots were programmed in 11.7% of the time). This is a crucially important observation: while Trustors

mention that bots do not need money (2.5%), nor do they have feelings (1.5%), they focus on the uncertainty of what *meaning* to attach to their Stage 1 decisions.

We now turn to examining the same frequencies over justifications among participants who believed the norm signal in Study 4 (Table S16). The comparison between justifications with the norm signal and without it is substantively important. When participants receive the norm signal, an additional 6.0%. of them think of consistency when the Helper is a bot, and an additional 10.0%. of them when the Beneficiary is a bot. While there is a slight shift (5.2%.) in the prominence of consistency in the human-only condition, the gap between this and other conditions in this regard shrinks. From these descriptive analyses, it appears that norm signals clarify the *meaning* of the behavior of Helpers. Last but not least, Trustors ponder the way bot Helpers make decisions, not their need of money or lack of feelings.

Code	Example answer
**1**	*Based on the past decision of Player 1 I felt like if the player 1 shared in the past they are likely to send money back if I send them money now in stage 2.*
**2**	*I chose to send 100 cents in each scenario. Even though Player 1 did not share in Scenario 1, I am taking a gamble that they will split the profit with me. Scenario 2 seems less risky as they chose to share in the first stage.*
**3**	*I tried to send the amount that would give us both equal bonus amounts or close to it.*
**4**	*I sent more money in the scenario that player 1 did share with the other player. I thought it was a good thing to reward generosity.*
**5**	*I just felt like I couldn't trust a bot to do the right thing and understand the situation.*
**5a**	*I didn't care to send any money to P1 because it's a bot. It's not going to get a bonus or be able to use one.*
**5b**	*I chose not to send any money to player 1 because they are a bot and I don't think that they would be sensitive to humans and feel the need to be fair by sending money back if I chose to send them something.*
**5c**	*Because player 1 is a computer, it feels more like rolling dice which I'm totally fine with.*
**5d**	*Being a bot I am still unsure how it will decide in this round.*
**6**	*Everyone on Turk needs money, most of us are on here because life didn't go how we thought it would. If me sending money helps someone else I want to do it so we can all earn and pay our bills.*
**7**	*Based on how generous he was.*
**8**	*I based it on how likely I thought he was to return anything. I also wanted to be the safest for my return on the game.*
**9**	*Based on if he shared or not.*
**10**	*I agreed with their decision about sharing with the Bot.*
**11**	*I do not want to risk losing my money voluntarily. I know I could be punished, but it is a risk I am willing to take.* In Stage 2, there is no Punisher; nor can the Helper they were paired with punish them when they "did not risk" any money.
**10a**	*Also, the rules for stage 2 said that player 4 was to be told how player 1 behaved in stage 1, which apparently is not the case?* The responded did not understand the strategy method, and appeared to be confused having to make two decisions.
**11b**	*I am trying to maximize my money, hopefully Player 1 will pick up on this, but I am reliant on him/her/bot.* Despite clearly signaling the identity of the Helper, this participant seems not to know this information.
**11c**	*I decided to keep my money no matter what. I have a suspicion the other MTurk worker isn't real.*
**12**	*I would in theory like to reward the one that shared, but at the same time I'm hoping that by sharing with the non-sharer, they will remember that kindness in the future.*
**13**	*100.*

Supplementary Table S14: **Typical answers of Turstors matching each code introduced.** Note that in the experiment the language of Beneficiary (Player 2), Helper (Player 1), Punisher (Player 3) and Trustor (Player 4) were avoided. E.g., Helpers may have shared more if Players 2 were called "Beneficiary" as it suggest that they "need" to benefit etc.

Justification (%)	No bot	Bot Helper	Bot Beneficiary
**1:** Consistency.	41.2	31.2	27.8
**2:** Assessing risk.	16.7	21.6	17.4
**3:** Higher level principle.	11.3	9.6	12.4
**4:** Rewarding/punishing the Helper.	8.2	4.3	6.0
**5:** Identity of the Helper.	0.6	12.3	1.7
**5a:** The Helper's need for money.	0.0	2.5	0.7
**5b:** The Helper's feelings.	0.0	1.5	0.3
**5c:** The Helper makes random decisions.	0.0	2.2	0.0
**5d:** Unclear how the Helper decides.	0.0	11.7	0.0
**6:** Wanting to help the Helper.	0.0	0.6	0.0
**7:** A characteristic of the Helper.	31.1	12.0	30.4
**8:** Maximize gain.	2.2	2.2	4.7
**9:** What the Helper did.	7.9	5.2	6.0
**10:** Who the Beneficiary is.	10.1	15.4	11.4
**11:** Confused Trustor.	3.5	4.3	2.7
**11a:** Misunderstood rules.	0.0	0.0	0.0
**11b:** Misunderstood who has what information.	0.0	0.0	0.0
**11c:** Did not believe the identities.	0.0	0.0	0.0
**12:** To influence the Helper's future actions.	0.0	0.0	0.3
**13:** None of the above.	3.4	4.3	2.7

Supplementary Table S15: **The distribution of reasons for making a decision by the Trustor in Study 4.** The first column contains the reasons, the second the share of times that a specific reason was mentioned in the human-only condition, the third the share of times that a specific reason was mentioned in the condition when the Helper was a bot, and the fourth the share of times that a specific reason was mentioned in the condition when the Beneficiary was a bot.

Justification (%)	No Bot	Bot Helper	Bot Beneficiary
**1:** Consistency.	46.8	37.3	37.8
**2:** Assessing risk.	15.3	19.6	21.6
**3:** Higher level principle.	14.4	12.8	12.2
**4:** Rewarding/punishing the Helper.	8.1	5.9	9.5
**5:** Identity of the Helper.	0.0	5.9	0.0
**5a:** The Helper's need for money.	0.0	4.9	0.0
**5b:** The Helper's feelings.	0.0	2.9	0.0
**5c:** The Helper makes random decisions.	0.0	1.0	0.0
**5d:** Unclear how the Helper decides.	0.0	15.7	0.0
**6:** Wanting to help the Helper.	1.8	0.0	0.0
**7:** A characteristic of the Helper.	33.3	14.7	32.4
**8:** Maximize gain.	5.4	2.0	5.4
**9:** What the Helper did.	10.8	19.6	10.8
**10:** Who the Beneficiary is.	0.0	1.0	17.6
**11:** Confused Trustor.	0.0	0.0	0.0
**11a:** Misunderstood rules.	0.0	0.0	0.0
**11b:** Misunderstood who has what information.	0.0	0.0	0.0
**11c:** Did not believe the identities.	0.0	0.0	0.0
**12:** To influence the Helper's future actions.	2.7	0.0	2.7
**13:** None of the above.	4.5	2.9	1.4

Supplementary Table S16: **The distribution of reasons for making a decision by the Trustor in Study 4 among those who believed the norm.** The first column contains the reasons, the second the share of times that a specific reason was mentioned in the human-only condition, the third the share of times that a specific reason was mentioned in the condition when the Helper was a bot, and the fourth the share of times that a specific reason was mentioned in the condition when the Beneficiary was a bot.

	No norm			Norm signal		
Justification	No Bot	Bot Helper	Bot Beneficiary	No Bot	Bot Helper	Bot Beneficiary
**1:** Consistency.	31.0	29.7	16.0	27.5	30.2	25.1
**2:** Assessing risk.	25.1	24.3	25.7	28.7	27.2	24.2
**3:** Higher level principle.	16.8	9.4	16.7	19.1	13.0	18.9
**4:** Rewarding/punishing the Helper.	8.6	5.7	5.7	6.8	5.6	8.1
**5:** Identity of the Helper.	0.0	18.3	1.0	1.6	16.8	2.2
**5a:** The Helper's need for money.	0.0	4.0	0.0	0.0	4.3	0.0
**5b:** The Helper's feelings.	0.0	2.0	0.0	0.0	0.4	0.0
**5c:** The Helper makes random decisions.	0.0	2.3	0.3	0.0	2.2	0.0
**5d:** Unclear how the Helper decides.	0.0	7.0	0.3	0.0	3.9	0.4
**6:** Wanting to help the Helper.	1.8	0.0	0.0	0.8	0.0	0.0
**7:** A characteristic of the Helper.	35.3	24.0	33.7	43.4	27.2	33.2
**8:** Maximize gain.	6.9	5.7	10.0	4.8	3.9	7.2
**9:** What the Helper did.	7.9	11.0	8.7	9.2	9.1	9.4
**10:** Who the Beneficiary is.	0.0	0.3	20.0	0.0	0.4	17.5
**11:** Confused Trustor.	0.3	0.0	0.0	0.8	0.4	0.4
**11a:** Misunderstood rules.	0.7	0.7	0.3	0.0	0.0	0.9
**11b:** Misunderstood who has what information.	0.0	0.0	0.3	0.0	0.0	0.4
**11c:** Did not believe the identities.	0.0	0.0	0.3	0.0	0.0	0.0
**12:** To influence the Helper's future actions.	0.3	0.0	0.0	0.4	0.4	0.4
**13:** None of the above.	3.6	4.7	5.0	1.6	2.2	4.0

Supplementary Table S17: **The distribution of reasons for making a decision by the Trustor in Study 5 among those who have not received the norm signal, and those who did and believed it.** The first column contains the reasons, the second and fourth the share of times that a specific reason was mentioned in the human-only condition, the third and fifth the share of times that a specific reason was mentioned in the condition when the Helper was a bot, and the fourth and sixth the share of times that a specific reason was mentioned in the condition when the Beneficiary was a bot.

We now turn to examining the same frequencies over justifications among participants in Study 5, displaying the answers of those who did not receive the norm signal, and those who did, and believed it (Table S17). The differences are similarly telling as in the comparison between Studies 2 and 4. Mentions of consistency increase when receiving the norm signal, so are those of the characteristic of Helpers. Importantly, Study 2 and 4 were coded by the same pair of coders, while Study 5 by another pair. Upon further inspection, the two pairs resolved ambiguity between "consistency" code and "Helper characteristics" differently, the first pair being more generous asserting a consistency code. Potentially similar coder differences underlie the discrepancy between studies in terms of assessing how risk-taking or risk-aversion influenced Trustors decisions. Importantly, in Study 5 as well Trustors focused on how bots make decisions (e.g., randomly, or unclear how) rather than bots' need for money or their feelings. Taken together, despite likely variability across coders, the same qualitative differences are born out in the data: norm signals solidify the *meaning* that Trustors attach to Helpers' actions.

Taken together, these justifications do not appear to refer to a commonly discussed perspective in the behavioral economics literature that decision makers, in this case, Helpers or Trustors playing with bots consider the people responsible for the bot's design, or the financial implications for giving up resources to the bot for the researchers. In fact, only one of the Helpers (and not a single Trustor) mentioned this perspective. Additionally, while confusion about the rules of the two-stage game is always a theoretical possibility, in our experiments few participants gave indication of such confusion (never more than 4% of the participants per experimental condition while being extremely generous with identifying such reasons by including answers that simply expressed that the participant did not believe the experimenter about the identities of players signaled). These observations further bolster our assertion that our interpretations anchored on signaling are likely to be correct in this context.

There are some clear limitations of the qualitative data. The use of the strategy method

consistent with the design of Jordan and colleagues (*3*) creates a different situation compared to asking participants to make one trust decision (when paired with a norm-following, and when paired with a norm-breaking Helper). This feature clearly influenced how participants reasoned. Additionally, since the study was survey-based, for the respondents who simply mentioned that a decision was made based on the *identity* of a player and mentioned that they were a bot, there were no options to ask the respondent to elaborate, specifically, if they thought of bots not needing money, not having feelings, or being unpredictable. Future work could be designed with the emerging themes documented here in mind, and with renewed emphasis on peoples' expectations over how bots make decisions.

Supplementary Note 9: Comparing the Distribution of Trust-gain in Study 2 and Study 3

In Study 2 the trust-gain is measured based on the trust decisions of the Trustor with real monetary stakes, while this measure in Study 3 is based on hypothetical decisions. Since the main goal of Study 3 is to correlate ones' perceptions about the norm-consensus in a given situation and the trust-gain based on trust-decisions, both of these measures need to be collected from the same individuals. However, having the same people participate in the strategic game, and then immediately answer norm-related questions would have likely yielded biased responses. Here, the main risk is that participants' decisions might have influenced their norm-consensus assessments; e.g., those acting selfishly could have "guessed" that there is no consensus over norms to manage their impression in front of the experimenter, thereby introducing a correlation consciously or unconsciously. To avoid such a confound, we measure norm-consensus information first, and then have participants make trust decisions. However, this induces a discrepancy between the design of Study 2 (real-stakes decisions) and Study 3 (hypothetical decisions). Given this, we here compare the distribution of the trust-gain in these studies using two measures: the Bhattacharyya coefficient and η, the overlapping index.

The Bhattacharyya coefficient (BC) is a measure of similarity between two discrete probability distributions p and q over the same domain X:

$$BC(p, q) = \sum_{x \in X} \sqrt{p(x)q(x)},$$

where values close to 1 suggest that two distributions are similar (with $BC = 1$, the distributions are identical), while values close to 0 indicate that the distributions are different.

The η is:

$$\eta(A, B) = \int_{\mathbb{R}^n} \min[f_A(x), f_B(x)]dx,$$

Experimental condition	η	Bhattacharyya coefficient
S-P1(P)-P2(P)-P3(P)	0.780	0.965
S-P1(B)-P2(P)-P3(P)	0.747	0.960
S-P1(P)-P2(B)-P3(P)	0.774	0.966
P-P1(P)-P2(P)-P3(P)	0.820	0.985
P-P1(B)-P2(P)-P3(P)	0.570	0.987
P-P1(P)-P2(B)-P3(P)	0.755	0.970
P-P1(P)-P2(P)-P3(B)	0.332	0.799

Supplementary Table S18: **Statistical comparison of the distributions of trust-gain in Study 2 and Study 3 by treatment**, using the η measure (*4*) and the Bhattacharyya coefficient (*5*). Each experimental condition is denoted by "S" that stands for sharing and "P" that stands for punishment that signals if the Helper (Player 1) or the Punisher (Player 3) is matched with the Trustor (Player 4) in the trust game, and the identity of participants in the third-party punishment game, either a "P," a person, or a "B," a bot in parenthesis after the role they fulfill: P1, P2, and P3 standing for Player 1, Player 2 and Player 3, respectively.

where $f_A(x)$ and $f_B(x)$ are two real probability density functions. The overlapping index η is $\mathbb{R}_n \times \mathbb{R}_n \to [0, 1]$, and the integral can be replaced by summation in the discrete case (including the present case). Similarly to the Bhattacharyya coefficient, η close to 1 indicates that the distributions are similar, while η close to 0 indicates the opposite.

The result of the comparison by experimental condition is represented visually in Figure S12, and numerically in Table S18. In this case the two measures generally agree (note slight differences in case of two of the punishment conditions), and suggest that the real-stakes and hypothetical decisions yielded similar distributions of the trust-gain.

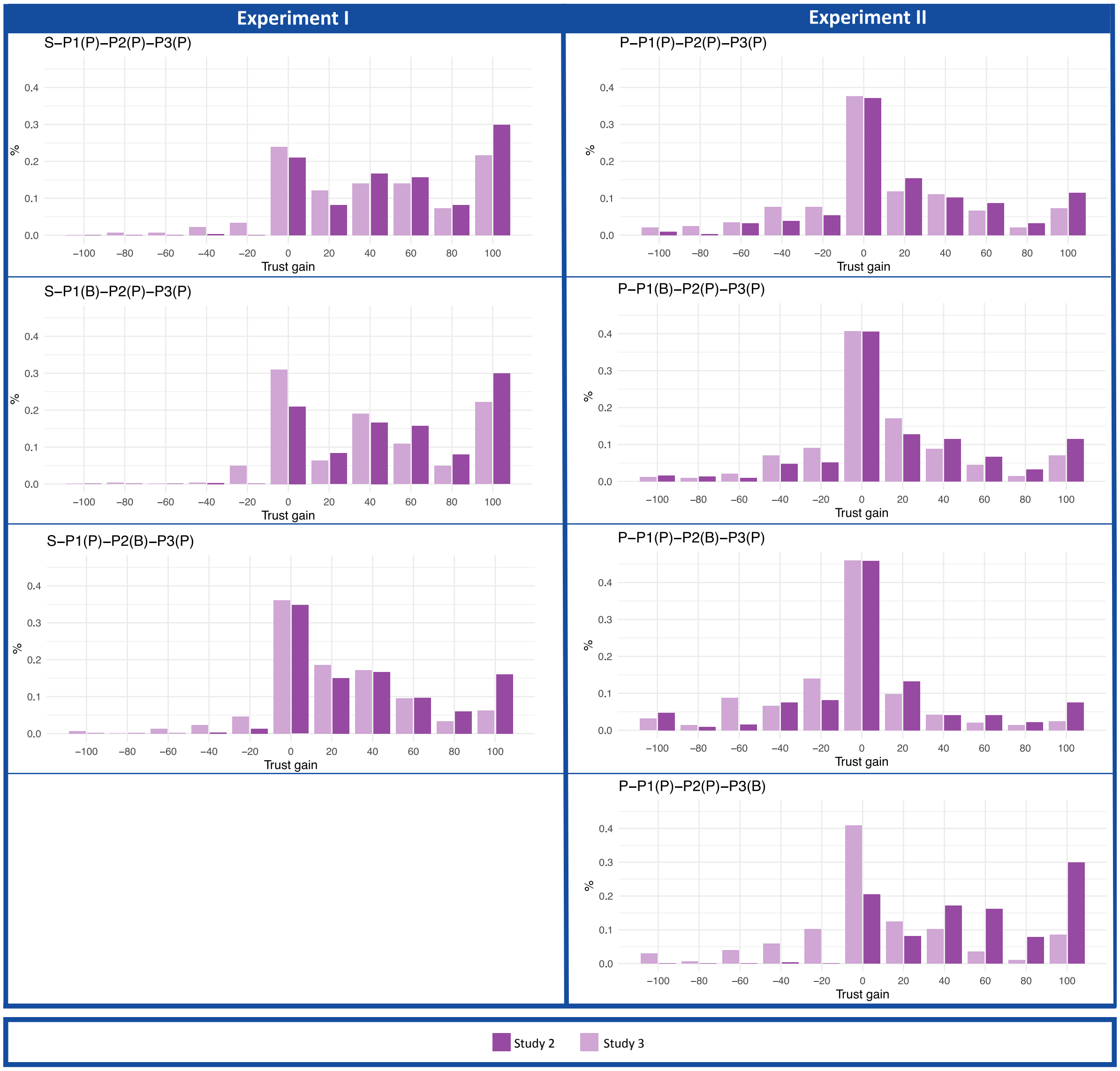

Supplementary Figure S12: **Comparing the distribution of trust-gain in Study 2 and Study 3.** Each experimental condition is denoted by "S" that stands for sharing and "P" that stands for punishment signaling if the Helper or the Punisher is matched with the Trustor in the trust game, and the identity of participants in the third-party punishment game, either a "P," a person, or a "B," a bot in parenthesis after the role they fulfill: P1 (Helper), P2 (Beneficiary), and P3 (Trustor), respectively.

Supplementary Note 10: Analyses of Selective Non-response

Prior research identified selective attrition as a threat to causal identification when effect-heterogeneity is present (*6*). Here, we test if the demographic composition of the sample who finished the study *after* passing the comprehension check questions, differs across experimental conditions (a more direct test would be to compare those that do not finish to those who do, but since the demographic characteristics were collected at the end of the survey we are unable to conduct such an analysis). It is possible that this selective attrition introduces bias if, for example, those with specific beliefs about interaction-norms in one condition left at disproportional rates over another. While this is not something we are able to observe, we do observe a number of demographic characteristics of participants, such as their age, race, income, education, gender and region. As long as these correlate with the unobserved variable in question we might get an indication that selective attrition poses a challenge to identification. We thus calculated the standardized mean differences (SMD) comparing the sample of participants in each experimental condition in every study to all other experimental conditions in the same study alongside all these variables, which allows us to look at the distribution of SMDs. In Study 5, we compare the experimental conditions with and without the norm manipulation. Fortunately, we found no systematic differences across the conditions in their demographic composition within study (SM Fig. S10–S13), and therefore it is unlikely that selective attrition compromises data quality and our inferences.

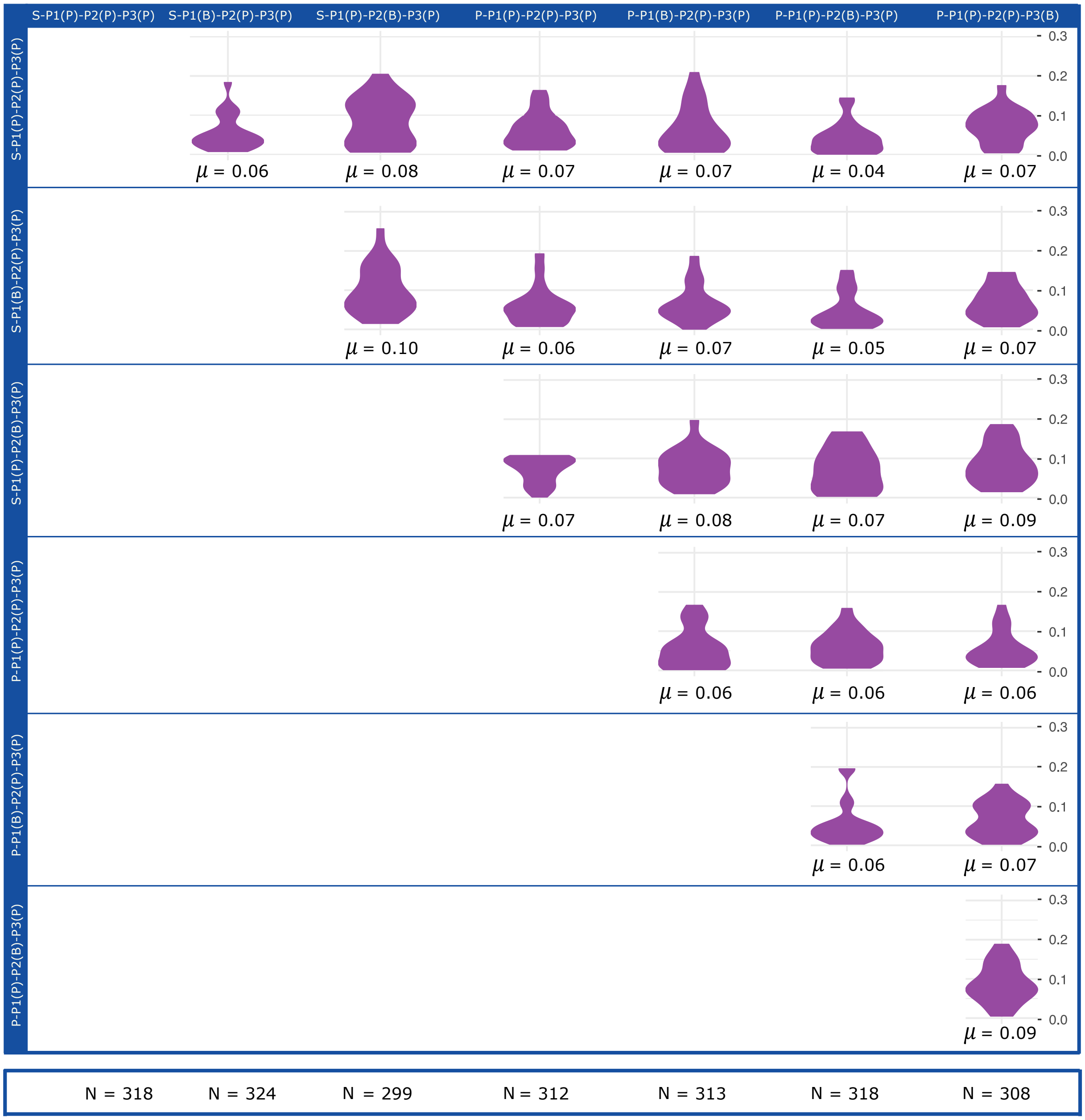

Supplementary Figure S13: **Pairwise demographic comparison of treatments in Study 2.** Each violin-plot represents the distribution of standardized mean differences (SMDs) for the demographic variables presented in Table S7 across the treatment indicated in the row and column; while μ indicates the mean of SMDs. Each experimental condition is denoted by "S" that stands for sharing and "P" that stands for punishment that signals if the Helper or the Punisher is matched with the Trustor in the trust game, and the identity of participants in the third-party punishment game, either a "P," a person, or a "B," a bot in parenthesis after the role they fulfill: P1 (Helper), P2 (Beneficiary), and P3 (Punisher), respectively.

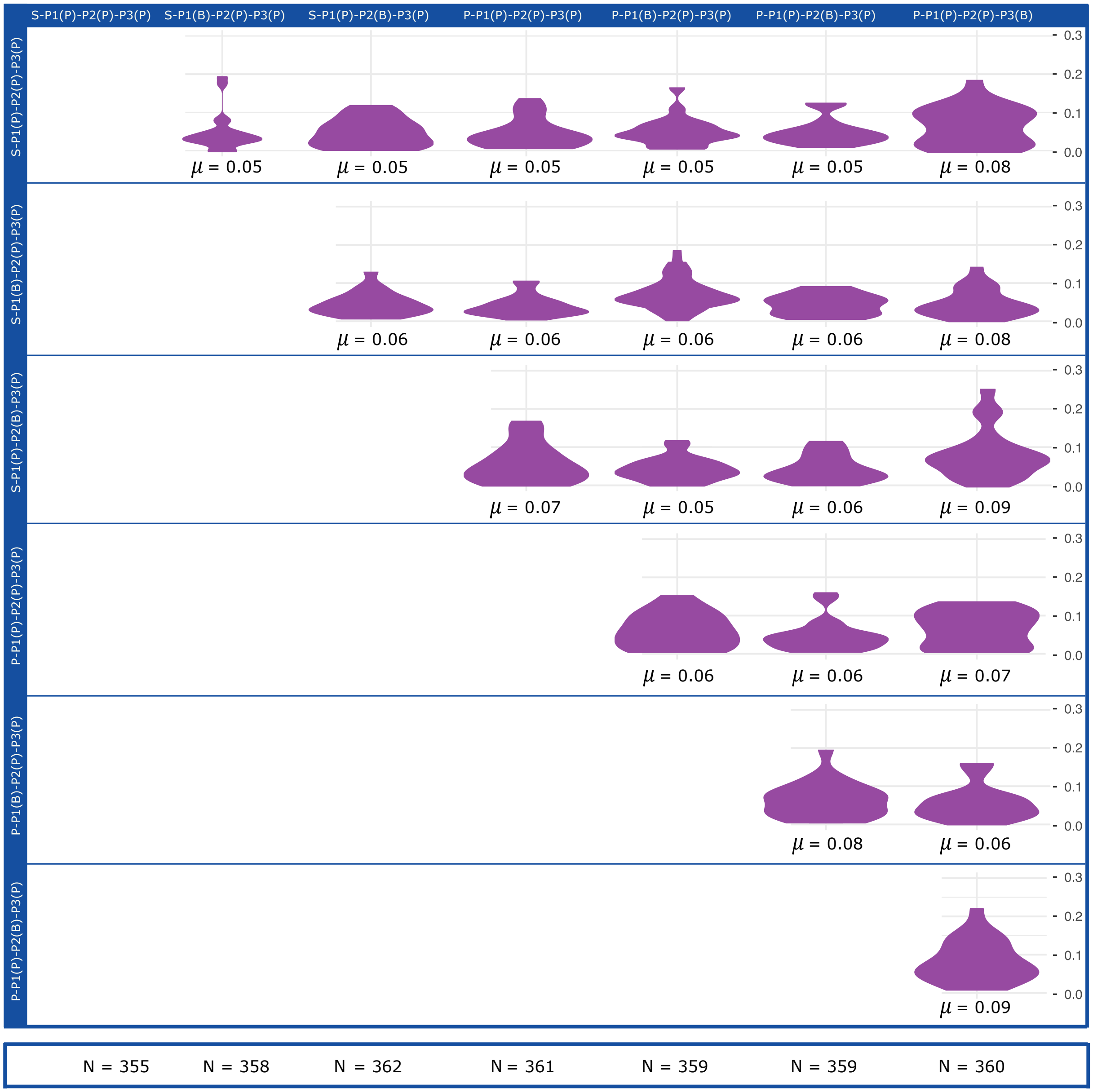

Supplementary Figure S14: **Pairwise demographic comparison of treatments in Study 3.** Each violin-plot represents the distribution of standardized mean differences (SMDs) for the demographic variables presented in Table S7 across the treatment indicated in the row and column; while μ indicates the mean of SMDs. Each experimental condition is denoted by "S" that stands for sharing and "P" that stands for punishment that signals if the Helper or the Punisher is matched with the Trustor in the trust game, and the identity of participants in the third-party punishment game, either a "P," a person, or a "B," a bot in parenthesis after the role they fulfill: P1 (Helper), P2 (Beneficiary), and P3 (Punisher), respectively.

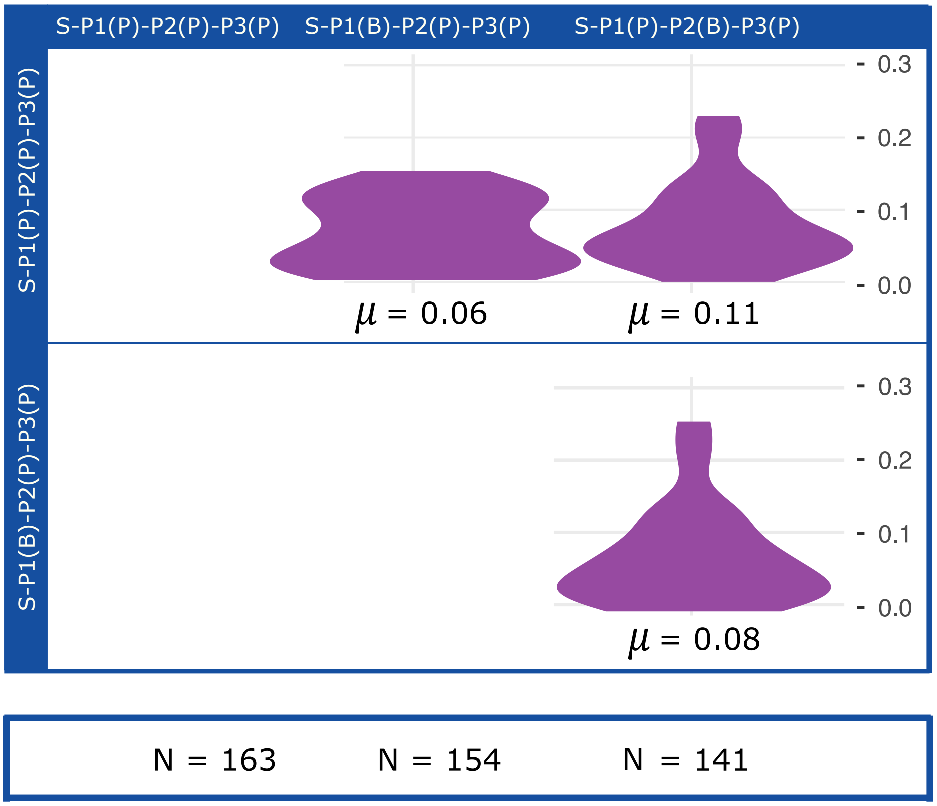

Supplementary Figure S15: **Pairwise demographic comparison of treatments in Study 4.** Each violin-plot represents the distribution of standardized mean differences (SMDs) for the demographic variables presented in Table S7 across the treatment indicated in the row and column; while μ indicates the mean of SMDs. Each experimental condition is denoted by "S" that stands for sharing and "P" that stands for punishment that signals if the Helper or the Punisher is matched with the Trustor in the trust game, and the identity of participants in the third-party punishment game, either a "P," a person, or a "B," a bot in parenthesis after the role they fulfill: P1 (Helper), P2 (Beneficiary), and P3 (Punisher), respectively.

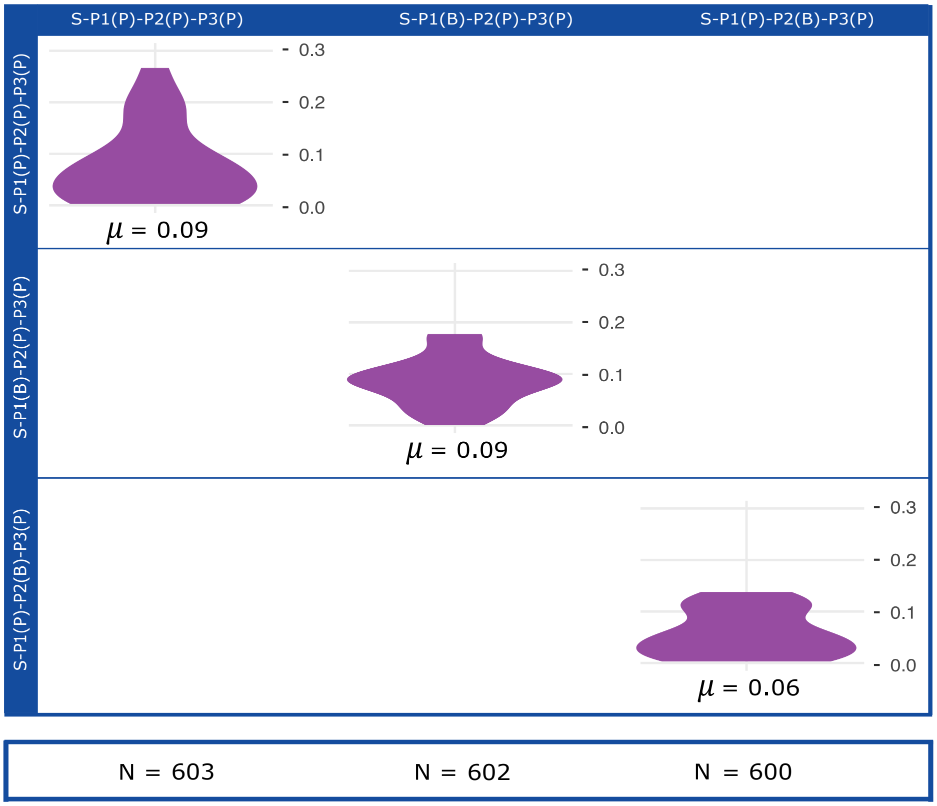

Supplementary Figure S16: **Within treatment demographic comparison with and without norm manipulation in Study 5.** Each violin-plot represents the distribution of standardized mean differences (SMDs) for the demographic variables presented in Table S7 within the same treatment across people exposed vs. not exposed to norm manipulation; while μ indicates the mean of SMDs. Each experimental condition is denoted by "S" that stands for sharing and "P" that stands for punishment that signals if the Helper or the Punisher is matched with the Trustor in the trust game, and the identity of participants in the third-party punishment game, either a "P," a person, or a "B," a bot in parenthesis after the role they fulfill: P1 (Helper), P2 (Beneficiary), and P3 (Punisher), respectively.

Supplementary References

1. Morris, S. B. & DeShon, R. P. Combining effect size estimates in meta-analysis with repeated measures and independent-groups designs. *Psychological Methods* **7**, 105–125 (2002).

2. Gibbons, R. D., Hedeker, D. R. & Davis, J. M. Estimation of effect size from a series of experiments involving paired comparisons. *Journal of Educational Studies* **18**, 271–279 (1993).

3. Jordan, J. J., Hoffman, M., Bloom, P. & Rand, D. G. Third-party punishment as a costly signal of trustworthiness. *Nature* **530**, 473–476 (2016).

4. Inman, H. F. & Bradley Jr, E. L. The overlapping coefficient as a measure of agreement between probability distributions and point estimation of the overlap of two normal densities. *Communications in Statistics—Theory and Methods* **18**, 3851–3874 (1989).

5. Bhattacharyya, A. On a measure of divergence between two statistical populations defined by their probability distributions. *Bulletin of the Calcutta Mathematical Society* **35**, 99–109 (1943).

6. Zhou, H. & Fishbach, A. The pitfall of experimenting on the web: How unattended selective attrition leads to surprising (yet false) research conclusions. *Journal of Personality and Social Psychology* **111**, 493–504 (2016).
