# Supplementary material for: Trust within human-machine collectives depends on the perceived consensus about cooperative norms
Source: Nat Commun. 2023 May 30;14:3108. doi: 10.1038/s41467-023-38592-5 (PMC10229533; doi:10.1038/s41467-023-38592-5)
Supplement: Supplementary file 2 — Reporting Summary [file 41467_2023_38592_MOESM2_ESM.pdf]

nature portfolio

Corresponding author(s): Talal Rahwan

Last updated by author(s): Apr 17, 2023

Reporting Summary

Nature Portfolio wishes to improve the reproducibility of the work that we publish. This form provides structure for consistency and transparency in reporting. For further information on Nature Portfolio policies, see our Editorial Policies and the Editorial Policy Checklist.

Statistics

For all statistical analyses, confirm that the following items are present in the figure legend, table legend, main text, or Methods section.

n/a	Confirmed	
☐	☒	The exact sample size (*n*) for each experimental group/condition, given as a discrete number and unit of measurement
☐	☒	A statement on whether measurements were taken from distinct samples or whether the same sample was measured repeatedly
☐	☒	The statistical test(s) used AND whether they are one- or two-sided<br>*Only common tests should be described solely by name; describe more complex techniques in the Methods section.*
☐	☒	A description of all covariates tested
☒	☐	A description of any assumptions or corrections, such as tests of normality and adjustment for multiple comparisons
☐	☒	A full description of the statistical parameters including central tendency (e.g. means) or other basic estimates (e.g. regression coefficient) AND variation (e.g. standard deviation) or associated estimates of uncertainty (e.g. confidence intervals)
☐	☒	For null hypothesis testing, the test statistic (e.g. *F*, *t*, *r*) with confidence intervals, effect sizes, degrees of freedom and *P* value noted<br>*Give P values as exact values whenever suitable.*
☒	☐	For Bayesian analysis, information on the choice of priors and Markov chain Monte Carlo settings
☒	☐	For hierarchical and complex designs, identification of the appropriate level for tests and full reporting of outcomes
☐	☒	Estimates of effect sizes (e.g. Cohen's *d*, Pearson's *r*), indicating how they were calculated

Our web collection on statistics for biologists contains articles on many of the points above.

Software and code

Policy information about availability of computer code

Data collection	Data collection was performed using Qualtrics available to the researchers via their academic institution.
Data analysis	Data analyses were performed by the R software (version: 3.6.3), which is open source. All code and data are available here: https://osf.io/jzpvs/. No custom codes have been developed, the standard R code for the figures/tables in the main are included in the repository.

For manuscripts utilizing custom algorithms or software that are central to the research but not yet described in published literature, software must be made available to editors and reviewers. We strongly encourage code deposition in a community repository (e.g. GitHub). See the Nature Portfolio guidelines for submitting code & software for further information.

Data

Policy information about availability of data

All manuscripts must include a data availability statement. This statement should provide the following information, where applicable:

- Accession codes, unique identifiers, or web links for publicly available datasets
- A description of any restrictions on data availability
- For clinical datasets or third party data, please ensure that the statement adheres to our policy

As stated in the submitted manuscript, data and code are deposited here: https://osf.io/jzpvs/ for purposes of reanalysis. The data we deposit does not include personally identifiable information, such as MTurk IDs of respondents as per the IRB protocol.

Research involving human participants, their data, or biological material

Policy information about studies with human participants or human data. See also policy information about sex, gender (identity/presentation), and sexual orientation and race, ethnicity and racism.

Reporting on sex and gender	Information about gender was collected, and is based on respondents' self-reports with three categories available specified on the survey: male, female and other. This variable was used to describe the sample, and was included as a control variable in regression models. The research did not develop hypotheses about sex and gender or heterogeneity of effects by sex/gender, therefore, no such investigation was performed. This variable is also part of the data set made available.
Reporting on race, ethnicity, or other socially relevant groupings	Information about race/ethnicity was collected and was established based on self-reports of respondents. The available categories for respondents were: American Indian or Alaska Native, Asian or Asian American, Black or African American, Hispanic or Latino/a, Middle Eastern or North African, White or Other. Respondents were allowed to select as many categories as they wished (i.e., these categories were non-exclusive). These descriptive statistics are reported, and in regression analyses a control variable of White (the majority in the US context), and non-White were used. The research did not develop hypotheses about race/ethnicity or heterogeneity of effects by race/ethnicity, therefore, no such investigation was performed. This variable is also part of the data set made available.
Population characteristics	See above.
Recruitment	As described, participants were recruited online via the MTurk platform. The sample is not representative of the American population; tends to be younger, more educated, more likely to identify as non-Hispanic White compared to the general population. Should these characteristics influence our findings in important ways they may not be generalized to the general population of Americans. In addition, selective attrition from treatment arms may introduce additional biases. We believe the impact of this is minimal as participants were motivated to finish the study in order to receive their compensation. In addition, we do not find evidence that attrition rates were widely varying across treatment arms, and have not found the demographic composition of respondents to be different across arms.
Ethics oversight	The study was approved by the NYU Abu Dhabi IRB (#062-2019), and informed consent was obtained from study participants consistent with the IRB protocol.

Note that full information on the approval of the study protocol must also be provided in the manuscript.

Field-specific reporting

Please select the one below that is the best fit for your research. If you are not sure, read the appropriate sections before making your selection.

☐ Life sciences ☒ Behavioural & social sciences ☐ Ecological, evolutionary & environmental sciences

For a reference copy of the document with all sections, see nature.com/documents/nr-reporting-summary-flat.pdf

Behavioural & social sciences study design

All studies must disclose on these points even when the disclosure is negative.

Study description	The manuscript reports results from five consecutive studies. All studies are quantitative experimental studies.
Research sample	All five studies were conducted using the subject pool of Amazon Mechanical Turk. As described, the following exclusion criteria were used to enter the studies: 95% approval rating (with the exception of Study 1 where this value was set to 90%), at least 100 HITs completed, US location, not on the universal exclude list, not from suspicious geo-locations (lists managed by CloudResearch, previously TurkPrime). Additional data quality filters, such as CloudResearch approved participants were used when available. Participants who completed Study 1 were 59% male (vs non-male including female and other), and 76% identified as as non-Hispanic White, with an average age of 38.2 (sd = 10.1). Participants who were allowed to complete Study 2 were 48% male (vs non-male including female and other), 75% identified as non-Hispanic White, with an average age of 37.1 (sd = 11.8). Participants in Study 3 were 50% male (vs non-male including female and other), 70% identified as non-Hispanic White, with an average age of 37.5 (sd = 12.6). Participants in Study 4 were 47% male (vs non-male including female and other), 75% identified as non-Hispanic White, with an average age of 40.5 (sd = 12.4). Finally, participants in Study 5 were 47% male (vs non-male including female and other), 75% identified as non-Hispanic White, with an average age of 41.3 (sd = 12.6). The sample is not representative to the American population; tends to be younger, more educated, more likely to identify as non-Hispanic White compared to the general population.<br><br>The convenience sample was used to recruit a large, geographically diverse sample for multiple experiments with a multitude of experimental conditions with the ability to distribute incentives to study participants effectively.
Sampling strategy	The studies build closely on the work of Jordan and colleagues (2016), and sample sizes were determined on the basis of that publication. No power calculations were conducted for this reason. A one-in-a-lifetime participation was allowed in Study 2 and 3, participants who took the study were randomly assigned to experimental conditions. Those who did not show adequate comprehension in Study 2 were not allowed to participate in that study. As for Study 4, only specific participants from Study 2 were invited as we follow a within-person design. As for Study 5 we allowed participants who took part previously to enter the study out of data quality considerations. Study 1, which was chronologically conducted the last allowed previous participants to enter.

	Study 1 does not use randomization. Study 2 and 3 use randomization. Respondents were blinded to the complete list of possible experimental conditions, although from the description they could have guessed other experimental conditions. Study 4 does not use randomization as we invited Study 2 participants who were assigned to the same experimental condition as in Study 2 in terms of the identity of their interaction partners, but received additional information about norm consensus. Study 5 follows a between person design with blinded randomization.<br><br>The convenience sample was used to recruit a large, geographically diverse sample for multiple experiments with a multitude of experimental conditions with the ability to distribute incentives to study participants effectively.
Data collection	Data collection was conducted online via Qualtrics. Participants were not informed of the full set of experimental conditions, they were only informed of the condition they took part in. As the data collection took place online, the researches were not present. We do not know if participants completed the survey alone.
Timing	Data collection for Study 1 took place between 7-15 of June 2022, for Study 2 between 8-13 of August, 2019; for Study 3 between 14-19 of July, 2020; for Study 4 between 3-17 of August, 2020; and for Study 5 between 11-28 of September, 2021.
Data exclusions	As previously described, to enhance data quality only specific MTurk workers saw the human intelligence task (see Research Sample section). In addition, we excluded from Study 2 respondents who did not show adequate comprehension (see Sampling Strategy) to ensure that respondents were aware of the consequences of the decisions they made. We conduct a variety of robustness analyses, including one where we exclude participants who did not recall correctly the identity of the other participants they were grouped with, which was communicated to them as part of the experiment. From Study 4 and Study 5 we excluded in our main analysis those who did not believe the norm-consensus manipulation, and included robustness checks on the full sample in the Supplementary Materials. These exclusions were applied because we can not randomize participants to beliefs, only stimuli, and those who did not believe the treatment they were assigned to may have behaved differently.
Non-participation	We conduct analyses of dropout. Specifically, after the participants saw the treatment they were assigned to an average attrition rate across treatments (based on the identity of those participants interacted with) of 2.53% in Study 2, 0.88% in Study 3, 1.35% in Study 4, and 0.01% in Study 5, respectively was observed, and is reported in the manuscript. As per our additional analyses, dropout appears to be random, and is likely a result of connectivity issues faced by participants.
Randomization	Study 1 does not have randomization. Participants were randomly assigned to experimental conditions in Study 2, Study 3 and Study 5 aiming to recruit a roughly equal number of participants in each experimental condition relevant for each study. Study 4 follows a within-person design, therefore participants who were recruited for Study 4 participated in the exact same condition as per their role and the identity of others in their groups that they took part in Study 2. In Study 5 we recruited a smaller number of Helpers and Punishers as our focus was on Trustors decisions.

Reporting for specific materials, systems and methods

We require information from authors about some types of materials, experimental systems and methods used in many studies. Here, indicate whether each material, system or method listed is relevant to your study. If you are not sure if a list item applies to your research, read the appropriate section before selecting a response.

Materials & experimental systems

n/a	Involved in the study
☒	☐ Antibodies
☒	☐ Eukaryotic cell lines
☒	☐ Palaeontology and archaeology
☒	☐ Animals and other organisms
☒	☐ Clinical data
☒	☐ Dual use research of concern
☒	☐ Plants

Methods

n/a	Involved in the study
☒	☐ ChIP-seq
☒	☐ Flow cytometry
☒	☐ MRI-based neuroimaging
